# Identifying Signatures of Natural Selection in Tibetan and Andean Populations Using Dense Genome Scan Data

**DOI:** 10.1371/journal.pgen.1001116

**Published:** 2010-09-09

**Authors:** Abigail Bigham, Marc Bauchet, Dalila Pinto, Xianyun Mao, Joshua M. Akey, Rui Mei, Stephen W. Scherer, Colleen G. Julian, Megan J. Wilson, David López Herráez, Tom Brutsaert, Esteban J. Parra, Lorna G. Moore, Mark D. Shriver

**Affiliations:** 1 Department of Anthropology, Pennsylvania State University, University Park, Pennsylvania, United States of America; 2 Max Planck Institute for Evolutionary Anthropology, Leipzig, Germany; 3 The Centre for Applied Genomics and Program in Genetics and Genomic Biology, The Hospital for Sick Children, Toronto, Ontario, Canada; 4 Department of Genome Sciences, The University of Washington, Seattle, Washington, United States of America; 5 Affymetrix, Inc., Santa Clara, California, United States of America; 6 Department of Molecular and Medical Genetics, Faculty of Medicine, University of Toronto, Toronto, Canada; 7 Department of Anthropology and Altitude Research Center, University of Colorado, Denver, Colorado, United States of America; 8 Department of Exercise Science, Syracuse University, Syracuse, New York, United States of America; 9 Department of Anthropology, University of Toronto, Mississauga, Ontario, Canada; 10 Departments of Public Health Sciences, Anthropology and Obstetrics-Gynecology, Graduate School of Arts and Sciences, Wake Forest University, Winston-Salem, North Carolina, United States of America; University of California Davis, United States of America

## Abstract

High-altitude hypoxia (reduced inspired oxygen tension due to decreased barometric pressure) exerts severe physiological stress on the human body. Two high-altitude regions where humans have lived for millennia are the Andean Altiplano and the Tibetan Plateau. Populations living in these regions exhibit unique circulatory, respiratory, and hematological adaptations to life at high altitude. Although these responses have been well characterized physiologically, their underlying genetic basis remains unknown. We performed a genome scan to identify genes showing evidence of adaptation to hypoxia. We looked across each chromosome to identify genomic regions with previously unknown function with respect to altitude phenotypes. In addition, groups of genes functioning in oxygen metabolism and sensing were examined to test the hypothesis that particular pathways have been involved in genetic adaptation to altitude. Applying four population genetic statistics commonly used for detecting signatures of natural selection, we identified selection-nominated candidate genes and gene regions in these two populations (Andeans and Tibetans) separately. The Tibetan and Andean patterns of genetic adaptation are largely distinct from one another, with both populations showing evidence of positive natural selection in different genes or gene regions. Interestingly, one gene previously known to be important in cellular oxygen sensing, *EGLN1* (also known as *PHD2*), shows evidence of positive selection in both Tibetans and Andeans. However, the pattern of variation for this gene differs between the two populations. Our results indicate that several key HIF-regulatory and targeted genes are responsible for adaptation to high altitude in Andeans and Tibetans, and several different chromosomal regions are implicated in the putative response to selection. These data suggest a genetic role in high-altitude adaption and provide a basis for future genotype/phenotype association studies necessary to confirm the role of selection-nominated candidate genes and gene regions in adaptation to altitude.

## Author Summary

High-altitude hypoxia is caused by decreased barometric pressure at high altitude, and results in severe physiological stress to the human body. Three human populations have resided at high altitude for millennia including Andeans on the Andean Altiplano, Tibetans on the Himalayan plateau, and Ethiopian highlanders on the Semian Plateau. Each of these populations exhibits a unique suite of physiological changes to the decreased oxygen available at altitude. However, we are just beginning to understand the genetic changes responsible for the observed physiology. The aim of the current study was to identify gene regions that may be involved in adaptation to high altitude in both Andeans and Tibetans. Genomic regions showing evidence of recent positive selection were identified in these two high-altitude human groups separately. We found compelling evidence of positive selection in HIF pathway genes, in the globin cluster located on chromosome 11, and in several chromosomal regions for Andeans and Tibetans. Our results suggest that key HIF regulatory and targeted genes are responsible for adaptation to altitude and implicate several distinct chromosomal regions. The candidate genes and gene regions identified in Andeans and Tibetans are largely distinct from one another. However, one HIF pathway gene, *EGLN1*, shows evidence of directional selection in both high-altitude populations.

## Introduction

As human populations migrated across the globe, they encountered numerous environments each with unique ecological conditions. These colonizers responded to the niche-specific environmental pressures both culturally and biologically. One such newly encountered environment was high altitude. High-altitude regions of the earth lie above 2,500 meters (m) sea level. The extreme environmental conditions experienced at high altitude challenge the ability of humans to live and reproduce, i.e., adapt and/or acclimatize. Some of the environmental hardships at high altitude include but are not limited to decreased ambient oxygen tension, increased solar radiation, extreme diurnal ranges in temperature, arid climate, and poor soil quality. Behavioral or cultural modifications buffer many of these factors. However, low ambient oxygen tension, caused by decreased barometric pressure and commonly referred to as high-altitude hypoxia, cannot readily be overcome by cultural buffers. Rather, physiological acclimatization and/or genetic adaptation is required for populations residing at altitude to overcome this environmental stress.

**Figure 1:**
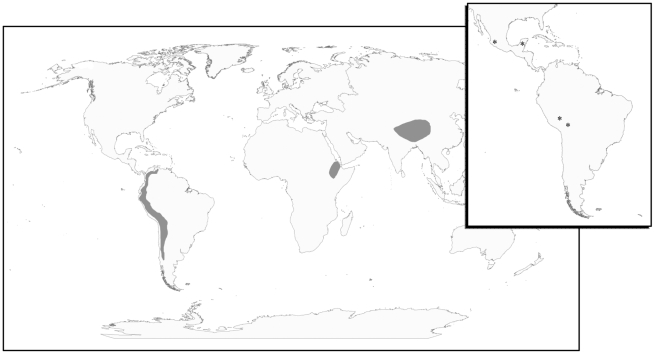
The geography of human adaptation to high altitude. Geographic locations where humans have adapted to life at high-altitude are indicated in grey and include the Andean Altiplano of South America, the Tibetan Plateau of Central Asia, and the Semien Plateau of Ethiopia. Only populations from the Andean Altiplano and the Tibetan Plateau were considered here. Inset: Map locations of the four Native American population samples including Peruvian Quechua, Bolivian Aymara, Nahua, Mixtec, and Tlapanec speakers from Guerrero, Mexico, and Maya from the Yucatan Peninsula, Mexico.

The Tibetan Plateau and the Andean Altiplano are two high-altitude regions where human populations have resided for millennia (Figure 1). According to archaeological data, they were first populated approximately 25,000 and 11,000 years ago, respectively [1], [2]. Today, the populations indigenous to these high-altitude zones possess unique suites of physiological characteristics with respect to one another and with respect to low-altitude populations (for review see [3]). Researchers have sought to understand the physiological differences between high- and low-altitude populations and whether such differences are the result of acclimatization or adaptation [4]. Several studies have shown that Tibetan populations exhibit lower than expected hemoglobin concentrations [5]–[7]. This is in contrast to Andean populations and to high-altitude sojourners who show elevated hemoglobin concentrations [5]. Other important differences concern the extent to which these lifelong high-altitude residents exhibit a blunted ventilatory response to acute hypoxia, their degree of protection from altitude-associated fetal growth restriction as well as their susceptibility to chronic mountain sickness and hypoxic pulmonary hypertension [4], [8], [9]. Related research has explored the heritability of specific altitude phenotypes such as arterial oxygen saturation and hemoglobin concentration [5], [10], [11]. One heritability study concluded that a major autosomal dominant locus exists for high oxygen saturation, where Tibetan women carrying this high oxygen saturation allele had a higher offspring survival rate than women possessing the low oxygen saturation allele [10]. Research of this nature documents the role of local adaptation, not simply acclimatization, to the high-altitude environment.

Even though the physiology of these populations has been well studied [3], [4], [12], [13], very little research has been devoted to the identification of the genes responsible for the observable physiological differences [14]. It is challenging to speculate from existing data what patterns of genetic variation underlie adaptation to altitude in Andeans and Tibetans. Studies identifying genetic variants associated with particular physiological phenotypes exhibit varying results. The lactase persistence phenotype is one example. Here functionally similar changes in the same gene, Lactase (*LCT*), have evolved independently in African and European populations to produce the same phenotypic outcome [15]. Genes affecting skin pigmentation show a different pattern from that observed for lactase persistence: separate genes are responsible for light-skinned phenotypes in East Asians and Europeans [16], [17]. Another well-studied human adaptive trait is malarial resistance. Like the pattern observed for skin pigmentation, multiple genes confer adaptive resistance to malaria in different populations [18]–[20] and like lactase persistence, particular mutations, namely the sickle cell S allele have recurred. Therefore, it is unclear whether Tibetan and Andean populations should be expected to show similarities or differences in genes or functionally different changes in the same genes that are responsible for their distinct high-altitude phenotypes. Moreover, it is possible that changes in different genes, but genes that are part of the same biochemical pathway, are responsible for the observable phenotypic differences between these two groups. For example, genes belonging to the hypoxia inducible transcription factor (HIF) pathway - important in embryogenesis, development, and homeostasis - and the renin-angiotensin system (RAS) are involved in oxygen sensing and metabolism. Variants identified in genes from these two pathways are known to affect particular altitude phenotypes [21]–[24]. For instance, the angiotensin converting enzyme (*ACE*) insertion-deletion (I/D) polymorphism has been significantly associated with the resting and exercise SaO_2_ among Quechua [23]. Additionally, genes in the alpha and beta globin gene family are involved in hemoglobin production. Accordingly, since such pathways or systems has been hypothesized to help regulate physiological responses to hypobaric hypoxia, they may be enriched for genes showing evidence of recent positive selection in high-altitude populations.

The goal of this study was to identify candidate genes for high-altitude adaptation based on signatures of positive selection in Andeans and Tibetans. Previously, we analyzed data from ∼500,000 SNPs to search for signatures of positive directional selection in Andeans [25]. This was the first such study of its kind. Here we increase the number of assayed SNPs to 906,600, and expand the populations to include a second high-altitude human group, Tibetans. We identified selection-nominated candidate genes and gene regions by two methods. First, we looked across each chromosome for extended regions of statistical significance for a given test statistic to identify candidate gene regions with previously unknown physiological functions with respect to altitude phenotypes. Second, we targeted genes that are members of biochemical pathways with known physiological responses to hypoxia including the HIF pathway, the RAS, and the globin family of genes [26]. In these two high-altitude groups we employed four test statistics commonly used to detect positive directional selection. Each test statistic possesses varying degrees of efficacy depending on the allelic background of the populations considered, the strength of selection, the type of variation natural selection acted upon (e.g., new mutations or standing variation), and the length of time elapsed since the start of the selective event. By comparing and contrasting two human populations who have adapted to life at altitude, we hope to better characterize the genetic mechanisms responsible for adaptation to high-altitude hypoxia, contribute to the understanding of the genetic and evolutionary architecture of adaptation to altitude, and provide deeper insight into the genes responsible for human phenotypic diversity.

## Results

We assayed 905,747 SNPs on the autosomes and X chromosome using the Genome-Wide Human SNP Array 6.0 by Affymetrix Inc. (Santa Clara, CA) to identify regions of the genome that have been subject to recent positive selection in two high-altitude groups, Andeans and Tibetans. In total, we assayed 49 ethnic Tibetans and 49 Andeans (25 Bolivian Aymara and 24 Peruvian Quechua). In addition to these two highland populations, we surveyed three lowland groups including 39 Mesoamericans, 60 Europeans, and 90 East Asians. The latter two populations correspond to the individuals included in the Haplotype Mapping project (HapMap) [27]. Sampling locations for each of the Native American samples are shown in Figure 1.

### Andean and Tibetan Population Stratification

**Figure 2:**
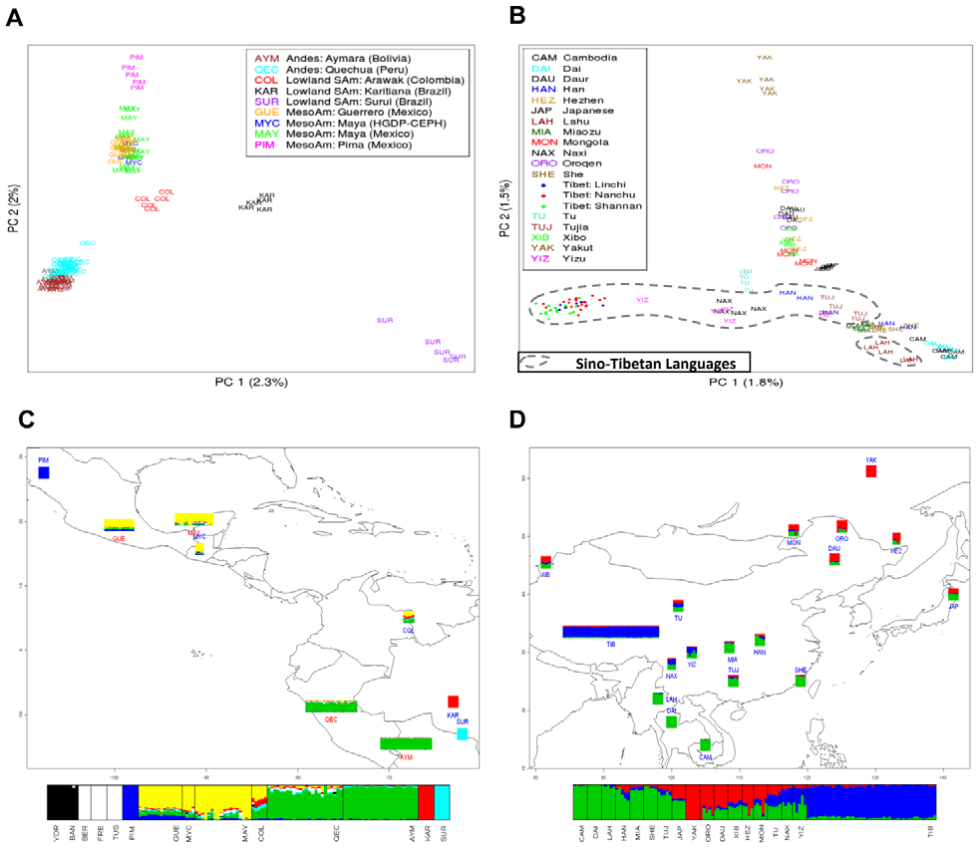
Individual ancestry estimates. (A) Indigenous American individual genetic structure using PCA with a set of 221,046 SNPs selected to remove the influence of European admixture (see Materials and Methods and Figure S1) Europeans have been removed to simplify the representation; (B) East Asian individual genetic structure using PCA with the full set of 439,046 SNPs; (C) *Frappe* map file showing Indigenous American and (D) East Asian population structure. Population abbreviations are as in Figure 2A and Figure 2B, respectively. Additional European and African populations are included in the *frappe* bar chart in Figure 2C, and include Yoruban (YOR), Bantu (BAN), Berber (BER), French (FRE), and Tuscan (TUS). Each individual is represented by a vertical line that is divided into colored segments representing the genomic contribution from a particular *K* ancestral cluster.

To better understand the genetic structure within these high-altitude populations and their relationship to low-altitude populations, we looked at population structure using the subset of SNPs that overlap between the Affymetrix SNP Array 6.0 and the Affymetrix SNP Array 5.0. This allowed us to compare Andeans and Tibetans to five individuals from each of the 51 Human Genome Diversity Project-Centre d'Étude du Polymorphisme Humain (HGDP-CEPH) population samples [28]. We performed a principle component analysis (PCA) to detect population structure and applied the maximum likelihood method as implemented in *frappe* to estimate individual ancestry proportions (see Materials and Methods section for details) [29], [30]. For the Andean and Tibetan PCA plots presented in Figure 2, the Tracy-Widom (TW) statistic was highly significant for PC1 through PC10 and the first two PCs had TW p-values largely below 10e^−10^.

The PCA plot depicted in Figure 2A illustrates that the Bolivians (Aymara) and Peruvians (Quechua) form a population cluster distinct from the other Indigenous American samples. A striking concordance with geographical location of the populations sampled is rendered in both the PCA plot (Figure 2A), with PC1 reflecting a North-South axis and PC2 an East-West axis, and the *frappe* map file (Figure 2C). The Colombian Arawak speakers appear at the genetic crossroads of Andeans, Brazilian Indigenes, and Mesoamericans, corresponding to their geographic location. Intriguingly, the Columbians appear more closely related to the Mesoamericans than to any of the other South American groups, which could be the result of genetic drift in these two regional groups.

The East Asian population analysis shows that genetic differentiation is highly correlated with language family (Kruskal-Wallis p<10e-18 for PC2) and geography (p = 2e-05, Mantel test between the geographic coordinates of each population sample and PC1-PC2 cluster centroid). The Tibetans form a distinct cluster separate from the other East Asian and Central Asian populations (Figure 2B and 2D). This may be the result of genetic drift within the Tibetan population; however, without a denser sampling of Himalayan populations this pattern is difficult to interpret. The Tu, Naxi, and Yizu (one individual in particular) show the strongest genetic affinity to the Tibetans. The Lahu, who speak a Tibeto-Burman language and are thought to have originated on the Tibetan plateau, cluster near their present-day geographic neighbors, the Dai and Cambodians (Figure 2B) and move towards the Tibetans in the PCA. Hence, despite a hit of genetic affinity for Tibet, the Lahu have assimilated largely with neighboring populations. To address the potential effect of the larger Tibetan sample size, we performed the PCA and the *frappe* analysis using equal numbers of Tibetan and HGDP-CEPH subjects. The same clustering effect is observed regardless of the Tibetan sample size included in the analysis (data not shown). In addition, we measured population structure within Tibet using the Kruskal-Wallis rank sum test on PC1 for the Tibetans versus their county labels. This analysis indicates that the Tibetans show within population substructure at the county level (p = 0.00036).

### Genomic Signatures of Positive Selection

We searched for signals of recent directional selection in Andeans and Tibetans separately to distinguish similarities and/or differences between these two highland groups' evolutionary response to high-altitude hypoxia. To identify selection-nominated candidate genes, we compared the patterns of variation between high- and low-altitude populations using four statistical tests; namely locus specific branch length (LSBL), the log of ratio of heterozygosities (ln*RH*), a modified Tajima's *D* statistic that considers *D* values in two populations termed the standardized difference of *D*, and the whole genome long range haplotype (WGRLH) test [31]–[34]. LSBL was calculated for each SNP in the dataset, whereas ln*RH* and the standardized difference of *D* were calculated for overlapping sliding windows 100 kilo base pairs (kb) in length with an offset of 25 kb. Statistical significance for each of the tests was determined using empirical, heuristic, p-values (P_E_ values) with the exception of the WGLRH test, which assessed significance using the gamma distribution as estimated using maximum likelihood methods. X chromosome data were analyzed separately from the autosomal SNP data because of the higher rates of evolution on the X chromosome relative to the autosomes. For both autosomal and X chromosome SNPs, we included only those for which the call rate was 95% or greater in the populations being considered for a given test statistic. For LSBL, we compared Tibetans to East Asians and Europeans and compared Andeans to Mesoamericans and East Asians. SNPs with less than a 95% call rate in all three populations were removed from the analysis. For ln*RH* and the standardized difference of *D*, SNPs with less than a 95% call rate in Andeans and Mesoamerican or Tibetans and East Asians were removed.

We looked across the genome to identify regions showing statistical evidence of recent positive selection in Andeans and Tibetans. SNPs or SNP windows falling in the top 5% or 1% of the empirical distribution generated for each test statistic were identified as statistically significant. Table 1 lists the significant SNP comparisons or SNP windows for each of the four test statistics applied to these data. Given the large number of statistically significant LSBL SNP comparisons, ln*RH* and standardized difference of *D* windows, chromosomal regions with clusters of significant test statistics were considered as strong candidates for positive selection. The hypergeometric distribution, calculated for 1 megabase non-overlapping windows along each chromosome, was used to identify extended regions of statistical significance for each test statistic. The p-value for each window was corrected for multiple tests using the Bonferroni correction. In total, p-values for 2,728 windows were calculated for each of the LSBL, ln*RH*, and the standardized difference of *D* statistics. Significant p-values were defined such that 1 false positive would be expected for all observed windows. Using this definition, windows where p≤0.004 were considered to be statistically significant.

**Table 1:** Significant SNPs or SNP windows in Andeans and Tibetans for P_E_≤0.05 and P_E_≤0.01.

Population	Test	Autosomes	P_E_ = 0.05	P_E_ = 0.01	X	P_E_ = 0.05	P_E_ = 0.01
Andean	LSBL	856,231	42,812	8,562	36,160	1,808	362
	ln*RH*	106,163	5,308	1,062	5,869	293	59
	*D*	106,109	5,305	1,061	5,862	293	59
	WGLRH	69,226	178	NA	271	0	NA
Tibetan	LSBL	845,054	42,253	8,451	36,031	1,802	360
	ln*RH*	106,140	5,307	1,061	5,869	293	59
	*D*	106,093	5,305	1,061	5,862	293	59
	WGLRH	79,938	436	NA	1046	2	NA

Autosomes and the X chromosome are listed separately.

Selection-nominated candidate regions were identified by looking for continuously significant one megabase regions of LSBL and the standardized difference of *D* or ln*RH* and standardized difference of *D*. Using these criteria, we identified 14 regions in Tibetans and 37 regions in Andeans (Table S1) as candidate chromosomal regions for high-altitude adaptation. In Andeans, several chromosomes exhibit two or more consecutive statistically significant one megabase windows including chromosomes 3, 7, and 12. The largest of these includes four consecutive regions of statistical significance on chromosome 12 ranged from 109,000,000 and 113,000,000. The first of the chromosome 12 regions, ranging from 109,000,000 to 110,000,000, is statistically significant for LSBL, ln*RH*, and the standardized difference of *D*. The remaining three regions, spanning 110,000,000 to 113,000,000, are statistically significant for LSBL and the standardized difference of *D*. In total, 47 genes are encoded by this 4 megabase window and include genes involved in immunity and cellular housekeeping among others (Table S2). In Tibetans, a single megabase window on chromosome 2 that spans 46,000,000 to 47,000,000 contains the HIF pathway candidate gene endothelial PAS domain protein 1 (*EPAS1 or HIF2a*). This region is significant for LSBL, ln*RH*, and the standardized difference of *D*. *EPAS1* is among the top candidate genes for the HIF pathway in this highland population (see next section). Of the gene regions identified, none overlap between Tibetans and Andeans. Thus, even if there has been convergent evolution at the phenotypic level, there is no evidence to suggest that the convergence is attributable to adaptive changes in the same set of genes.

In addition to identifying genomic regions, we also looked specifically at candidate loci for evidence of recent positive selection. We focused on 2 pathways, the HIF and the RAS, as well as the globin family of genes as each has been hypothesized to be involved in adaptation to altitude. The results for each are discussed below.

### Signatures of Selection in the HIF Genes

In Andeans, 42, 17, 13, and 3 HIF pathway genes including 50 kb upstream and downstream of the start and end coordinates of each gene were statistically significant (p≤0.05) for LSBL, ln*RH*, the standardized difference of *D*, and the WGLRH test, respectively. The corresponding numbers in Tibetans were 36, 13, 17, and 1 HIF genes. The total number of SNPs and SNP windows for the HIF pathway are shown in Table 2. Tables S3 and S4 list the total number as well as the number of significant SNPs and SNP windows for each candidate gene in Andeans and Tibetans.

**Table 2:** Total LSBL SNPs, ln*RH* windows, or the standardized difference of *D* windows analyzed for the HIF pathway, RAS, and globin family.

		Andean			Tibetan		
Pathway	Genes	LSBL	ln*RH*	Tajima's *D*	LSBL	ln*RH*	Tajima's *D*
HIF	75	3168	719	718	3135	719	710
RAS	11	431	54	53	423	45	53
Globin	27	1240	189	193	1239	184	193

**Figure 3:**
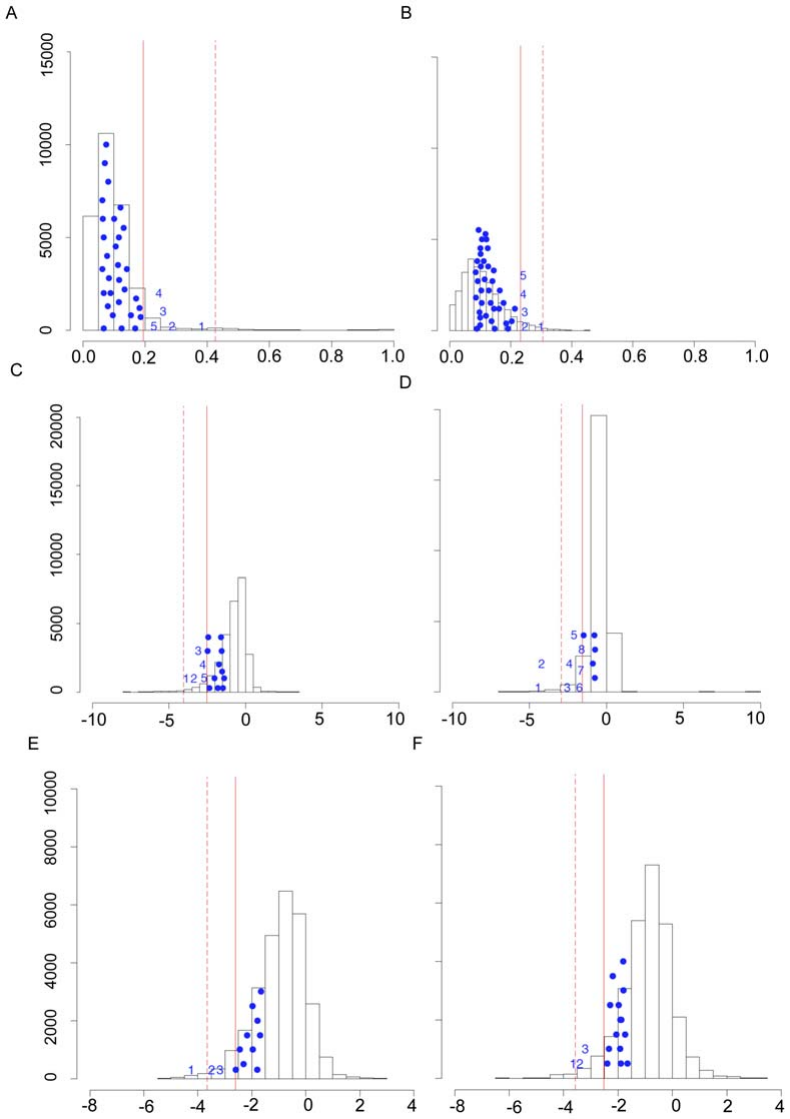
HIF pathway candidate genes showing evidence of positive directional selection in Andeans and Tibetans. The most significant test statistics for the HIF pathway candidate genes are plotted against the null distribution generated by plotting the highest ranked (i.e. most significant) test statistic for each gene from the dataset for LSBL in (A) Andeans and (B) Tibetans, for ln*RH* in (C) Andeans and (D) Tibetans, and for the standardized difference of *D* for in (E) Andeans and (F) Tibetans. The solid line indicates the 5% empirical cut off and the dashed line indicates the 1% empirical cut off for each distribution. The maximum test statistic for each of the HIF pathway candidate genes that had one or more SNPs falling in the top 5% of the empirical distribution is plotted on the figure as a solid circle. Those SNPs that fall in the top 5% of the empirical distribution and that are significant under the null distribution shown here for each population are plotted as numbers. In Figure A, 1 = *EGLN1*, 2 = *NOS2A*, 3 = *TGFA*, 4 = *CXCR4*, and 5 = *PRKAA1*. For Figure B, 1 = *EPAS1*, 2 = *EPO*, 3 = *CASR*, 4 = *EDN1*, and 5 = *EGLN1*. For Figure C, 1 = *ELF2*, 2 = *IL1A/IL1B*, 3 = *TNC*, 4 = *FRAP1*, 5 = *POLR2A*. For Figure D, 1 = *PIK3CB*, 2 = *COPS5*, *3 = EGLN1*, *4 = VEGFC*, *5 = IL1B*, *6 = EPAS1*, *7 = RBX1*, *8 = IL1A*. In Figure E, 1 = *PRKAA1*, 2 = *NOS2A*, 3 = *EDNRB*. For Figure F, 1 = *EPAS1*, 2 = *ARNT*, 3 = *ADRA1B*.

To gain a clearer understanding of the HIF pathway genes showing evidence of recent positive selection, we ranked and prioritized the candidate genes against a null distribution generated using a subset of the data. To do so, the most significant SNP or SNP window for LSBL, ln*RH*, or the standardized difference of *D* was identified in each statistically significant *HIF* gene. These values were compared against a null distribution generated by plotting the most significant SNP or SNP window in each gene of the dataset for LSBL, ln*RH*, or the standardized difference of *D* (Figure 3) [35]. Five, five, and three HIF pathway candidate genes were identified in Andeans for LSBL, ln*RH*, and the standardized difference of *D* respectively. For the Tibetans, five, eight, and three HIF pathway candidate genes were identified for LSBL, ln*RH*, and the standardized difference of *D* respectively. Both protein kinase, AMP-activated, alpha 1 catalytic subunit (*PRKAA1*) and Nitric Oxide Synthase 2A (*NOS2A*) are significant for LSBL and the standardized difference of *D* in Andeans. Tibetans exhibit significant LSBL, ln*RH*, the standardized difference of *D* values for a single gene, *EPAS1*. No gene is significant for both ln*RH* and the standardized difference of *D* in Andeans.

**Figure 4:**
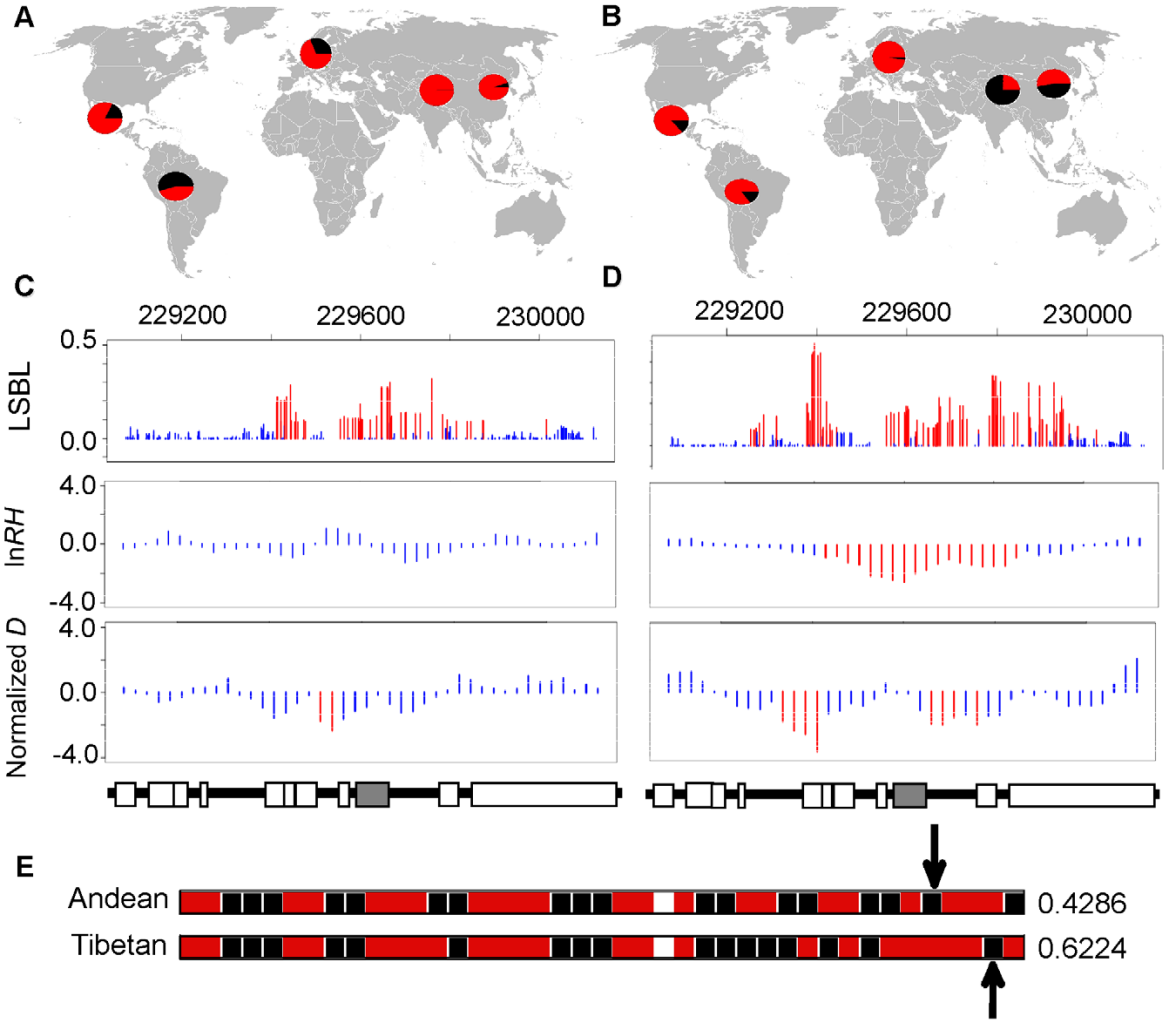
Genetic variation at *EGLN1*. The distribution of allele frequencies is shown for the two *EGLN1* SNPs, rs1769792 and rs12030600, exhibiting the highest ranked LSBL values in Andeans (A) and Tibetans (B), respectively. The derived G allele is shown red and the ancestral A allele is shown in black for rs1769792. The derived A allele is shown in red and the ancestral G allele is shown in black for rs12030600. LSBL, ln*RH*, and standardized difference of *D* are plotted for *EGLN1* including 500 kb upstream and downstream from the gene in (C) Andeans and (D) Tibetans. Significant SNPs or sliding windows are shown in red (P_E_<0.05) whereas non-significant SNPs or SNP windows are depicted in blue. The genes found in the plotted region are shown below the plots. *EGLN1* is indicated in grey. The most frequent haplotype in the 100 kb region surrounding *EGLN1* is depicted for Andeans and Tibetans (E). Ancestral alleles are depicted in black and derived alleles are depicted red. The ancestral/derived state for a single site is unknown and is depicted as an unfilled box. The population frequency is listed to the right of each haplotype. The arrows indicate the location of the SNPs displaying the most extreme LSBL values plotted in 4A and 4B.

One of the most striking results is the signature of positive selection found for the gene egl nine homolog 1 (*EGLN1 or PHD2*) in both Andeans and Tibetans. In Andeans, 25 of the 40 *EGLN1* SNPs and in Tibetans 28 of 39 SNPs are significant at the 5% level for LSBL. Table 3 enumerates the empirical rank and P_E_ values for each of the statistically significant *EGLN1* SNPs in both Andeans and Tibetans. Andeans display six SNPs within the top 1,000 SNPs of the entire empirical distribution, with rs1769792 ranking the highest of these six SNPs (Figure 4A). Tibetans exhibit only a single SNP in the top 1,000 SNPs of the empirical distribution, rs12030600 shown in Figure 4B, but display 13 SNPs significant at p≤0.01. In addition, both populations display extended regions of significant statistics 500 kb upstream and downstream of *EGLN1*, but the pattern differs for each population. Andeans exhibit a concentration of high LSBL values, extended regions of negative ln*RH*, and standardized difference of *D* (Figure 4C). For the Tibetan population, many of the SNP comparisons show high LSBLs coupled with extended regions of significant ln*RH* and standardized difference of *D* values (Figure 4D). When considering the haplotype structure in this region, both Andeans and Tibetans show a single dominant haplotype in the 100 kb region surrounding *EGLN1*. This haplotype is unique to each population as shown in Figure 4E. Finally, pairwise *F*_ST_ was calculated between Andeans and Tibetans at distances of 100 kb and 500 kb surrounding the gene. The pairwise *F*_ST_ estimates between these two populations are 0.206 and 0.262, for 100 kb and 500 kb, respectfully, further illustrating that the patterns of selection observed in each population are distinct. This lack of haplotype sharing is consistent with both selection on shared standing variation and selection on new mutations that occurred after the two populations had separated. Additional work including genotype-phenotype associations and functional data will be necessary to identify the causal variant(s) and to distinguish if a shared variant(s) or independent mutations were selected for in these two highland groups.

**Table 3:** Empirical distribution rank and P_E_ value for each significant *EGLN1* SNP in both Andeans and Tibetans.

Population	rsID	LSBL	Rank	P_E_	Population	rsID	LSBL	Rank	P_E_
Andean	rs1769792	0.300	297	0.0003	Tibetan	rs12030600	0.223	506	0.0006
	rs1769813	0.272	604	0.0007		rs480902	0.185	1328	0.0016
	rs1339896	0.272	605	0.0007		rs2749710	0.183	1359	0.0016
	rs1765811	0.272	606	0.0007		rs16854388	0.181	1411	0.0017
	rs2355865	0.272	607	0.0007		rs2486729	0.171	1807	0.0021
	rs1614148	0.261	781	0.0009		rs2244986	0.171	1808	0.0021
	rs508618	0.183	4518	0.0053		rs2486731	0.159	2509	0.003
	rs12093061	0.120	18686	0.0218		rs2486746	0.156	2701	0.0032
	rs1769795	0.120	18715	0.0219		rs2739513	0.156	2702	0.0032
	rs2486746	0.116	20629	0.0241		rs2024878	0.156	2704	0.0032
	rs2790859	0.114	21483	0.0251		rs2790882	0.156	2705	0.0032
	rs961154	0.110	23333	0.0272		rs2066140	0.150	3186	0.0038
	rs2486736	0.108	24219	0.0283		rs2486732	0.143	3925	0.0046
	rs2066140	0.106	25464	0.0297		rs2437150	0.139	4326	0.0051
	rs2739513	0.106	25465	0.0297		rs1538664	0.118	8307	0.0098
	rs2024878	0.106	25469	0.0297		rs1769795	0.107	11409	0.0135
	rs2486731	0.106	25470	0.0297		rs2790859	0.107	11481	0.0136
	rs2749710	0.104	26577	0.031		rs12093061	0.106	11658	0.0138
	rs2437150	0.104	26959	0.0315		rs508618	0.098	14644	0.0173
	rs2790882	0.102	28319	0.0331		rs961154	0.096	15539	0.0184
	rs2486729	0.100	29207	0.0341		rs1765805	0.096	15598	0.0185
	rs2244986	0.100	29208	0.0341		rs1769792	0.083	22758	0.0269
	rs1538664	0.098	30987	0.0362		rs1339896	0.083	23213	0.0275
	rs480902	0.091	35927	0.042		rs1765811	0.083	23214	0.0275
	rs2486732	0.089	37900	0.0443		rs2355865	0.083	23215	0.0275
						rs1614148	0.083	23216	0.0275
						rs1769813	0.083	23313	0.0276
						rs7542797	0.064	40605	0.0480

In addition to identifying selection-nominated candidate genes, we also tested whether multiple genes in the HIF pathway have been subject to positive selection. To do so, we employed a Kolomorgorov-Smirnov (K-S) test to determine if the LSBL distribution of HIF pathway genes deviates from that of the non-HIF genes. We used a one-sided K-S test to compare the LSBL distributions of HIF versus non-HIF LSBLs. We found that the two distributions were not significantly different from one another, thus indicating that the HIF distribution is not enriched for SNPs falling in the 1% or 5% tail of the empirical LSBL distribution in Andeans (D_n,m_ = 0.0081, p = 0.6681) or Tibetans (D_n,m_ = 0, p = 1). This result suggests that HIF pathway genes have not evolved in concert, but rather that key HIF genes are involved in the adaptation to altitude in these two highland groups.

### Signatures of Selection in the Globin and RAS Genes

**Figure 5:**
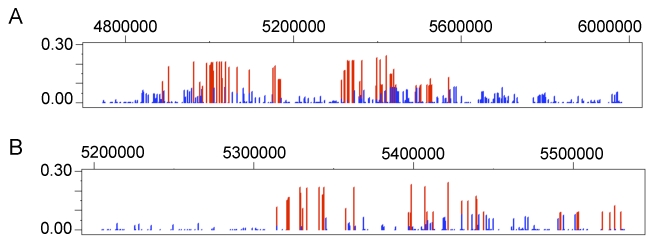
Genetic variation at *HBE1*. LSBL plotted for (A) 50 kb and (B) 500 kb surrounding *HBE1*. LSBL is shown on the X-axis and genomic position along chromosome 12 is shown on the Y-axis, with the axis labels located on the upper portion of the plots.

For the globin family of genes, one gene, *HBE1*, which is part of the beta globin gene cluster found on chromosome 11, contains 35 of the 82 significant Andean LSBL SNP comparisons (or 43%), identified for this gene system (Figure 5A). This suggests that *HBE1* has been subject to recent positive selection in Andeans. Twenty-eight and 8 windows containing globin genes are statistically significant for ln*RH* and the standardized difference of *D*, respectively. No 500 kb extended haplotype regions encompass a globin gene for the WGLRH test. Using the same re-sampling technique applied to the HIF genes, *HBE1* and *SATB1* fall in the top 1% of the null distribution for LSBL and top 5% for the standardized difference of *D*, respectively. No globin genes are statistically significant for ln*RH* nor do any genes show statistical significance for both LSBL and the standardized difference of *D*.

In the Tibetans, 113 statistically significant LSBL SNP comparisons in 13 genes contain a SNP in or within 50 kb upstream or downstream of a globin gene. For ln*RH*, ten windows containing SNPs from five globin genes are significant at α = 0.05. Five windows containing SNPs in *NFE2* are significant for standardized difference of *D*. The total number and the number of significant SNPs or SNP windows for each gene in the globin family are listed in Tables S3 and S4. No extended haplotype regions were identified for a globin gene. In comparison to the null distribution, nuclear factor (erythroid-derived 2) (*NFE2*) is statistically significant (p≤0.05) for both LSBL and the standardized difference of *D*. A second gene, myoglobin (*MB*), is statistically significant (p≤0.05) for LSBL. A previous study looking at sequence variability of the myoglobin gene (*MB*) in Tibetan populations did not find evidence of natural selection [36]. In this genome scan, the single significant SNP is located 27 kb upstream of the *MB* initiation codon. None of the SNPs within the coding region of this gene or other SNPs within 500 kb of *MB* are significant. Our findings taken together with the previous work suggest that the regulatory region may be involved in high-altitude adaptation, but that variation in the coding region of this gene does not show evidence of positive selection. Finally, as seen in the Andeans, no globin SNP is statistically significant for ln*RH*.

Evidence of positive selection in the RAS for both high-altitude groups is weak. Ten and 37 RAS SNPs display statistically significant LSBLs for Andeans and Tibetans respectively. Three ln*RH* windows, all containing SNPs in the gene angiotensin II receptor-associated protein (*AGTRAP*) are statistically significant for Andeans. No ln*RH* windows containing RAS SNPs are significant for Tibetans. None of the standardized difference of *D* windows falling in the bottom 5% for this statistic encompasses a RAS gene in Andeans. In the Tibetan population, eight significant standardized difference of *D* windows representing 3 genes contain RAS genes. No 500 kb extended haplotype regions contained a RAS gene region in either population. Likewise, no RAS gene falls in the top 5% or 1% with respect to the null distribution generated using the re-sampling technique described above for the HIF pathway candidate genes.

### Copy Number Polymorphisms

Common copy number polymorphisms (CNPs) and rare copy number variants (CNVs) were analyzed for evidence of recent positive selection in addition to the SNPs [37], [38]. As is the case with the SNPs, the CNPs analyzed here are unlikely to be the targets of directional selection. Rather, they are simply polymorphic sites of known genomic location that can be used to identify patterns of genetic variation associated with directional selection in highlanders compared to lowlanders [39]. CNPs were defined as the subset of CNVs that segregate in greater than 3% of the population, whereas rare CNVs were considered as present in less than 3% of the population. The analysis of CNPs revealed 1,316 common variants, 653 of which were biallelic and therefore included in the population genetic analysis. None of the biallelic CNPs that were statistically significant for LSBL or the relative haplotype homozygosity (REHH) calculated using SWEEP (see Materials and Methods section for details) overlapped with a HIF, RAS, or globin gene (Tables S5 and S6). We identified 1,207 rare CNVs in Andeans and Tibetans. By cross-referencing the CNVs with the selection-nominated candidate genes identified using the dense SNP data, we identified three Tibetan and one Andean CNV overlapping with extended haplotype homozygosity regions (Table S7). For Andeans, a single CNV on chromosome six overlapped with a significant hypergeometric region identified using LSBL and the standardized difference of *D*. This CNV was identified in a Bolivian individual and a Mexican individual. No Tibetan CNVs overlapped with any of the regions identified using the hypergeometric distribution.

## Discussion

In this study, we identified genomic regions showing evidence of recent positive selection in two high-altitude human populations, Andeans and Tibetans, using dense multilocus SNP genotype data. Putative natural selection candidate loci were detected in particular pathways with hypothesized roles in high-altitude adaptation as well as chromosomal regions with previously unknown involvement in altitude phenotypes.

Four tests based on different characteristics of the data were used in our analysis: LSBL, ln*RH*, the standardized difference of *D*, and the WGLRH test. It is worthwhile to review some important issues related to the characteristics of each of these statistics and their application to these data. LSBL is based on Wright's *F*_ST_ (see Materials and Methods) and summarizes the change in SNP allele frequency across three populations to identify loci displaying large frequency differences between populations. The value of LSBL will be contingent upon the initial frequencies of the alleles in the populations, the time since the populations diverged, and the strength of the selective event. Fixation of alternative alleles will result in the maximum LSBL. Such a scenario can occur regardless of whether selection operated on standing variation or on a new mutation. However, a maximum LSBL need not be observed to infer evidence of selection. In fact, intermediate LSBL values can be observed for loci under selection. Ln*RH* summarizes the reduction in heterozygosity in one population relative to another population. Thus, it identifies directional selection in the population with a loss of heterozygosity. Interestingly, ln*RH* can miss a signal of directional selection if the change in allele frequency is symmetrical about the midpoint in heterozygosity (changing from 40% to 60% allele frequency for example). Tajima's *D* uses the site frequency spectrum to detect departures from neutrality. In this instance, it was used to identify regions of the genome that have an excess of rare variants to detect positive selection. Lastly, the WGLRH test looks at patterns of LD to identify genomic regions that exhibit longer than expected LD given their frequency in the population. However, the WGLRH test only considers derived alleles with respect to an outgroup (e.g., chimpanzee) whose frequencies have risen to >0.85 in the populations under consideration. The problem with only considering haplotypes with high derived allele frequencies is that natural selection could also act to select the ancestral allele and these signatures would not be detected using the WGLRH test. The strength of selection, time since selection began, as well as the recombination background of the selected region will all affect the signal obtained when applying this statistic to a dataset. Given the aspects of genetic variation summarized by these statistics, it is not expected that the results of each of these tests will be significant. Rather, these statistical tests should be considered as complementary tests that can be useful for the identification of regions under positive selection.

Commonly, a traditional parametric-model approach is taken in screens for natural selection where the level of variability at candidate loci is compared to either simulated distributions or theoretical expectations [40], [41], [42]. However, recent research has shown that the empirical distribution may be better than a simulated or theoretical distribution because the latter two approaches may be confounded by underlying demographic assumptions [43]. By comparing individual SNPs to the genome-wide empirical distribution for each test statistic, the results of this study were not confounded by demography. Yet, the empirical distribution approach is not without problems. For example, consider a population where much of the allele frequency change across the genome was the result of genetic drift. In this scenario, the entire LSBL distribution would be shifted to the right. Higher levels of variance in LSBL and higher genome average LSBL levels could result in overlooking outliers that resulted from positive selection.

The chromosomal regions showing either extended significant regions for the standardized difference of *D* and LSBL or ln*RH* are excellent candidates for further study. Of the 52 regions identified, the four consecutive regions on chromosome 12 in Andeans and the single region on chromosome 2 containing the HIF pathway candidate gene *EPAS1* in Tibetans are of keen interest. When considering the genes encoded by the chromosome 12 region in Andeans, none stand out as obvious candidates for high-altitude adaptation. Further characterization of this chromosomal region will be necessary to elucidate the genetic variants responsible for the observed pattern. In Tibetans, the hypergeometric region containing *EPAS1* is a strong candidate for adaptation to altitude given the known biological function of *EPAS1* in oxygen sensing. Future genotype-phenotype correlation studies should focus their attention on this gene in Tibetans. In addition to the 52 chromosomal regions, the candidate regions identified by the WGLRH test are also excellent candidates for further study. Of these candidate regions, one Andean region contained a *HIF* pathway candidate gene, *TH*.

Given the divergence time of Andeans and Tibetans, their independent adaptation to high altitude, and their unique physiological adaptations to altitude, we find the overlap in the *EGLN1* signal to be of keen interest. *EGLN1* is part of the HIF pathway wherein cellular oxygen homeostasis is regulated by HIF-1. This heterodimer is composed of an α subunit and a β subunit. The β subunit is constitutively expressed whereas the α subunit is transcriptionally controlled by cellular O_2_ concentration [44], [45]. HIF-1α is a basic-helix-loop-helix protein encoded by the gene *HIF1A* [44], [46]. This protein mediates transcriptional responses to hypoxia in nearly 100 genes to control cellular oxygen supply and maintain cell viability during periods of low oxygen concentration. *EGLN1*, along with *EGLN2* (*PHD1*) and *EGLN3 (PHD3)*, is a molecular oxygen sensor that regulates the HIF transcriptional pathway [47]. In normoxia, *EGLN1* hydroxylates HIF-1α's oxygen dependent degradation domain which targets this protein for breakdown by the E3 ubiquitin ligase complex [48]–[50]. Decreases in oxygen tension lead to a reduction in prolyl hydroxylation of HIF-1α by *EGLN1*, thus increasing HIF levels and permitting HIF1 to continually target downstream genes to maintain cellular oxygen homeostasis [45]. Thus, *EGLN1* plays a critical role in cellular oxygen sensing. Our results suggest that adaptation has occurred independently at this gene in these two highland groups, although it is difficult to discern if selection operated on shared standing variation or new mutations.

In addition to *EGLN1*, the *HIF* pathway genes exhibiting the most compelling evidence of positive directional selection in Andeans are *PRKAA1* and *NOS2A*. *PRKAA* is a heterotrimeric enzyme belonging to the ancient 5′-AMP-activated protein kinase gene family involved in regulation of cellular ATP [reviewed in (49)]. *PRKAA1* functions as a cellular energy sensor under ATP-deprived conditions such as those experienced in hypoxia, thus suggesting a biologically-plausible role for the *PRKAA1* (AMPKa1)-mTOR pathway in metabolic responses to hypoxic environments. Also, it has been demonstrated that the *PRKAA1* gene product is essential for hypoxia-inducible factor-1 (*HIF-1*) transcriptional activity. *HIF-1* trans-activates multiple genes in the *HIF* pathway that are important for oxygen delivery [50]. In addition, *HIF-1* is critical for both embryonic vascularization and development. Therefore, if a genetic variant in *PRKAA1* contributes to the differential survival of babies at altitude, one would expect that the selection coefficient for this allele to be strong.

*NOS2A*, in combination with additional nitric oxide synthase isoforms, synthesizes nitric oxide (NO) from arginine and oxygen. NO is a signaling molecule with myriad physiological functions throughout the body. Important with regard to high-altitude adaptation, this gene is responsible for the production of NO, formerly known as endothelium-derived relaxing factor (EDRF), in endothelial and other cell types [51]. NO, in combination with a cascade of additional circulating substances prompts arterial smooth muscle relaxation, vasodilation and increased blood flow [52]. Erzurum *et al.* [51] have shown that NO production is increased in Tibetans resident at 4,200 m compared to sea-level controls. Moore and co-workers have shown that increased blood flow to the uteroplacental circulation is an especially important factor in protecting Tibetan as well as Andean high-altitude residents from altitude-associated reductions in fetal growth [52]–[54]. On balance, these studies suggest that vascular factors, not simply hematological or ventilatory systems, are critical for altitude adaptation in Tibetan and Andean populations. Here, we show preliminary evidence of positive selection in *NOS2A* in the Andean population, but do not show compelling evidence of positive selection in Tibetans. Both *PRKAA1* and *NOS2A* were identified in a previous study as selection-nominated candidate genes in Andeans using a subset of the data [25].

A single gene, *EPAS1*, exhibited a strong signature of recent positive selection in the Tibetan population. This was evident from the HIF pathway candidate gene analysis as well as the chromosomal scan. *EPAS1* is a HIF regulatory gene encoding a transcription factor that induces downstream genes when cellular oxygen levels decrease. Recently, this gene has been implicated in high-altitude pulmonary edema (HAPE) in Tibetan populations, although the results are not conclusive [55]. Further research elucidating the genotype-phenotype relationship between this gene and corresponding high-altitude phenotypes will be an important step in understanding the functional significance of *EPAS1* variation.

In summary, we performed a genome scan on high- and low-altitude human populations to identify selection-nominated candidate genes and gene regions in two long-resident high-altitude populations, Andeans and Tibetans. Several chromosomal regions show evidence of positive directional selection. These regions are unique to either Andeans or Tibetans, suggesting a lack of evolutionary convergence between these two highland populations. However, evidence of convergent evolution between Andeans and Tibetans is suggested based on the signal detected for the HIF regulatory gene *EGLN1*. In addition to *EGLN1*, a second HIF regulatory gene, *EPAS1*, as well as two HIF targeted genes, *PRKAA1* and *NOS2A*, have been indentified as selection-nominated candidate genes in Tibetans (*EPAS1*) or Andeans (*PRKAA1*, *NOS2A*). *PRKAA1* and *NOS2A* play major roles in physiological processes essential to human reproductive success [56]. Thus, in addition to demonstrating the likely targets of natural selection and the operation of evolutionary processes, genome studies also have the clear potential for elucidating key pathways responsible for major causes of human morbidity and mortality. Based on the findings of this study, it will be important to confirm the results with genotype-phenotype association studies that link genotype to a specific high-altitude phenotype.

## Materials and Methods

### Populations and Genome-Wide Data

High-density multilocus SNP genotype for 347 individuals were generated using the Genome-Wide Human SNP Array 6.0 by Affymetrix Inc. (Santa Clara, CA). This array includes 1.8 million genetic markers consisting of 906,600 SNPs located throughout the genome (SNPs: 869,225 on autosomes, 37,000 on the X-chromosome including 478 on the pseudo-autosomal region, 257 on the Y-chromosomes and 119 mitochondrial DNA SNPs). These genetic markers are typed on two arrays, named for the restriction enzymes used in the complexity reduction step of the reaction, the *NSP* array and the *STY* array. In total, 905,747 autosomal and X chromosome SNPs were analyzed. The Y-chromosome and mitochondrial DNA SNPs were not considered. In addition to SNPs, common copy number polymorphisms (CNPs) and rare number variants (CNVs) were included in this analysis. For this analysis, we considered common copy number polymorphisms (CNPs) as the subset of copy number variants that segregate in greater than 3% of the population, whereas CNVs that were found in less than 3% of the population were considered as rare CNVs. All CNPs and CNVs were called using Birdsuite (Cambridge, MA), and McCarrol *et al.'s* [57] high-resolution map of CNPs was used to define these loci (that is, McCarrol *et al.'s* [57] CNP map was derived using the same array resolution for the Affymetrix SNP 6.0 microarray as in our study). Next, CNVs were verified using two additional algorithms for CNV detection. They include the Affymetrix Genotyping Console (Santa Clara, CA) and a hidden markov model (HMM) from Partek Genomics Suite (St. Louis, MO). Stringent CNV calls were defined as CNVs that were detected by two or more algorithms. Therefore, in order for a CNV to be included in our analysis, it must have been detected by two of the three algorithms.

We assayed two high-altitude human populations, Andeans and Tibetans, as well as closely related low-altitude control populations to identify selection-nominated candidate genes or gene regions. These two populations were analyzed separately and the results from the independent analyses compared. The Andean sample was composed of 49 individuals belonging to two high-altitude populations and included 25 individuals of largely Aymara ancestry collected in La Paz (3,600 m), Bolivia, and 24 Peruvian Quechua from Cerro de Pasco (4,338 m), Peru [54], [58], with the Peruvian and Bolivian populations being sampled by two of us (LGM and TDB) and the Tibetans by one of us (LGM). The Tibetan sample consisted of 49 ethnic Tibetans from three counties within the Tibetan Autonomous Region of China, and can be broken down into 22, 20, and seven individuals from Nachu County (4,400 m), Shannan County (3700 m), and Linchi County (3,000 m), respectively [59]. The low-altitude control samples included four Mesoamerican populations including 25 Maya from the Yucatan Peninsula of Mexico (10 m), and 14 Mesoamericans composed of 2 Nahua, 7 Mixtec, and 5 Tlapanec speakers from Guerrero, Mexico (1,600 m). All Mesoamerican individuals were from populations that are not known to have lived at high altitude. In addition, we analyzed two HapMap Project (www.hapmap.org) populations consisting of 60 individuals from the United States of northern and western European ancestry (CEU), and 90 East Asians from Beijing (55 m), China and Tokyo (8 m), Japan. The population samples typed using the Affymetrix Inc. (Santa Clara, CA) Genome-Wide Human SNP Array 6.0 are listed in Table S8. Participants provided informed, written consent according to the guidelines approved by the Institutional Review Board at Penn State University. Genetic ancestry estimates were calculated for all of the Indigenous American individuals (both Andeans and Mesoamericans) using a panel of ancestry-informative markers (AIMs) that is useful for distinguishing between West African, Northern European, and Indigenous American populations [60], [61]. All the individuals included in this study show high levels of Indigenous American genetic ancestry (>90%) and lower components of West African and Northern European ancestry (<10%). No AIMs are currently available to distinguish Tibetan ancestry from Han Chinese ancestry. However, all Tibetan samples were collected from individuals living at least 20 kilometers from the nearest town to ensure minimal admixture with Han Chinese. All assayed samples were included in the SNP analysis, but for the CNV analysis, 16 samples, eight Tibetan, two Andean, and six Mesoamerican, did not pass the quality control filters and were removed from further CNV study.

### Population Structure Analysis

The EIGENSOFT package [29] was used for PC analysis, with default parameters, and nsnpldregress = 0. The “snpweightoutname” option was implemented to obtain the influence that each SNP weighs on the PC1 that separated the Europeans and Indigenous Americans (Figure S1A). We then trimmed the tails of the distribution of weights to obtain reduced SNP sets that bear less influence of SNPs informative for European/Indigenous American differences. For example, at a threshold of 0.9 (240,969 SNPs) the gap between Europeans and Indigenous Americans is partially reduced (in fact this axis switches from PC1 to PC2). At threshold 0.8 (Figure 1A) and below (data not shown), the European cluster is positioned in the midst of a consistent pattern of Indigenous American clusters.

Admixture estimates were performed with the maximum likelihood method implemented in *frappe* [30] that considers each individual's genetic makeup to be composed of *K* ancestral populations that sum to 1. All *frappe* runs were performed until the convergence criterion was met (less than 1 point of likelihood increase between each step). *Frappe* includes a stochastic aspect that can cause the results with the same input parameters to differ across runs. We have presented *frappe* results at the highest *K*-clustering that were consistent across multiple runs. For Figure 2C, we chose *K* = 7 (equivalent to *K* = 5 because we only mapped the non-European, non-African samples), as it was the highest *K* giving consistent clustering across 3/4 of the runs (the 4th run showed a split between Peruvian Quechua and Bolivian Aymara). For Figure 2D, we chose *K* = 3 because it was extremely consistent across 4 runs, whereas *K* = 4 separate the Lahu in 3/4 of the cases (the 4th run showing internal variation within Tibet). *Frappe* clustering for values of *K* other than those presented in Figures 2C and 2D are shown in Figures S2 and S3 (patterns beyond the chosen *K* are not the same across runs, although they appear recurrently in different forms across runs (not shown)).

Related individuals were removed based on IBD PI_HAT values (PLINK software) [62]. Two Maya samples appeared to cluster among the HGDP-CEPH individuals (HGDP00863 and HGDP00872). Five pairs of individuals (1 Bolivian and 4 Maya, pairs) appeared strongly related (PI_HAT>0.5) and one person of each pair was removed in subsequent structure analyses. Similar criteria lead to the removal of three Tibetan individuals. In order to avoid inter-population biases in missing data, we defined a threshold of at least three genotypes called per population sample. We also cleaned the merged dataset of SNPs with global minor allele frequency <1%. Merging our samples with HGDP-CEPH samples was performed with the PLINK software v1.05 [62], [63].

### Genome-Wide Analysis of Signatures of Positive Selection

In order to identify signatures of positive selection specific to Andeans and Tibetans, the Mesoamerican sample and the East Asian sample were used as low-altitude control populations for the Andeans while the European and East Asian samples were used as low-altitude control populations for the Tibetan population. Candidate positive selection loci were identified in Andean and Tibetan populations by applying four tests of natural selection. They include the locus-specific branch length (LSBL), the log of the ratio of heterozygosities (ln*RH*), the standardized difference of *D*, and whole genome long range haplotype (WGLRH) tests [31], [32], [33], [34]. LSBL, ln*RH* and the standardized difference of *D* were computed by implementing Perl scripts written specifically for this data set. The LSBL was computed for each SNP in the data set individually whereas an overlapping sliding windows approach was taken to calculate ln*RH* and the standardized difference of *D* for each window. A window size of 100 kb was selected based on the genome coverage and the marker density of the Affymetrix SNP Array 6.0. Statistical significance for each of the LSBL, ln*RH*, and the standardized difference of *D* statistics was determined by using its respective genome-wide empirical distribution generated by these data independently for the autosomal chromosome SNPs and X-chromosome SNPs. Separate analysis of X-chromosome markers was required given the smaller effective population size and the higher degree of natural selection observed and expected for markers on this chromosome. The empirical p-value for LSBL, ln*RH*, and the standardized difference of *D* was calculated by using the following equation:
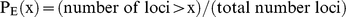



Those loci with P_E_ values falling in the top (LSBL) or bottom (ln*RH* and the standardized difference of *D*) 5% of the empirical distribution for the autosomal chromosomes or the X chromosome were considered statistically significant (α = 0.05). The WGLRH test was computed using the algorithm developed by Zhang *et al.* [32]. This test is based on the observation that loci in linkage disequilibrium (LD) with the functional SNP will be swept to fixation or near fixation during the selective event, resulting in haplotypes with high population frequencies coupled with long range LD. For this test, significance was assessed by comparing the relative extended haplotype homozygosity (REHH) of a specific core haplotype to the gamma distribution. The false discovery rate approach was then applied to correct for multiple tests [64].

In our study, LSBL was used to describe the relationship between the relevant populations at each locus by apportioning the genetic diversity into three population branches for each of the two population triangulations of interest. The population triangulations include 1) Andean, Mesoamerican and East Asian and 2) Tibetan, East Asian and European. To calculate LSBL, we computed Wright's *F*_ST_ using Weir and Cockerham's equation at every SNP position for each two-way population comparison (i.e., East Asian to Mesoamerican, East Asian to Andean, and Mesoamerican to Andean for the Andean triangulation) [65], [66]. Next, the pairwise *F*_ST_ values were used to calculate the LSBL as previously described [33], again at each SNP. With three contrasting populations, the LSBL statistic is mathematically equivalent to the population-specific *F*_ST_ introduced by Weir and colleagues in 1984 [66]. LSBL values falling in the upper 5% tail of the empirical distribution for the Andean population or the Tibetan population are suggestive of positive natural selection in that particular population.

The natural log of the ratio of the heterozygosity between two populations of interest, or ln*RH*, was used to summarize the extent to which population-specific loss in genetic diversity characterizes SNP regions and candidate genes in the two high-altitude populations under consideration [67], [68]. This statistic was calculated for each two-way population comparison using an overlapping sliding window size of 100,000 base pairs (bp) and moving in 25,000 bp increments along a chromosome. For the high-altitude populations, we focused our search on the Andean-Mesoamerican ln*RH* values for the Andean panel or the Tibetan-East Asian ln*RH* values for the Tibetan. Statistically significant negative ln*RH* values for each of these comparisons indicate regions where there has been a reduction in variation in the population of interest that did not occur in the closely related comparison population.

Regions of the genome with negative Tajima's *D* values are a hallmark of positive selection. However negative values of *D* can also result from demographic events, specifically the recovery from a population bottleneck. As for the ln*RH* analysis, Tajima's *D* was calculated for each population using an overlapping sliding window size of 100 kb with a 25 kb offset. We used a modification of the Tajima's *D* statistic, standardized Tajima's *D* to compare Tajima's *D* across windows. This statistic is similar to the i*Hs* statistic [69] and is calculated using the following equation:
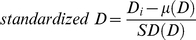
Where *D_i_* is the Tajima's *D* calculated for a sliding window in a given population panel (Andean, Mesoamerican, or East Asian), *μ* is the mean Tajima's *D* for all windows, and *SD* is the standard deviation of Tajima's *D* for all windows. Using this statistic, we identified significantly negative windows in Andean and Tibetan populations. However, because we are interested in the identification of regions of the genome that have been subject to recent positive selection in Andeans and Tibetans, we compared Tajima's *D* in Andeans vs. Mesoamericans, as well as Tibetans vs. East Asians. To do so, we used the standardized difference of *D* to summarize the difference of Tajima's *D* between two populations using the equation:
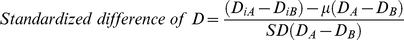
Here, *D_i_*_A_ is Tajima's *D* computed for a given sliding window in population A, *D_i_*_B_ is Tajima's *D* computed for a given sliding window in population B, *μ* is the mean Tajima's *D* for all windows, and *SD* is the standard deviation of Tajima's *D* for all windows. Again, Tajima's *D* was calculated for each population using an overlapping sliding window size of 100 kb with a 25 kb offset. Therefore, we first identified negative Tajima's *D* values in Andeans or Tibetans using the normalized Tajima's *D* statistic. We then applied the standardized difference of *D* metric to identify windows that were significantly different between high- and low-altitude human groups.

The final test used to infer positive selection was the WGLRH test of Zhang *et al.* [32]. This test first calculates the REHH for each core haplotype in the data set and identifies core haplotypes with longer than expected ranges of linkage disequilibrium (LD) given their frequency in the population. A gamma distribution is then estimated using maximum likelihood methods against which the REHH of each core haplotype is tested to determine if its respective p-value is suggestive of recent, positive selection. This test then considers the ancestral state of the alleles, determined by a closely related outgroup, to identify SNPs where the derived allele has risen to extremely high frequencies (>0.85). For this data set, the ancestral state for all SNPs available in the chimpanzee sequence was retrieved using the UCSC genome browser. In total, the ancestral states for 846,032 SNPS on the autosomes and X chromosome were obtained. Lastly, the WGLRH test applies a false discovery rate approach to control for false positives and identifies significant extended haplotypes using the gamma distribution.

To identify CNPs that may be involved in adaptation to high altitude in Andeans or Tibetans, we compared population frequencies for each biallelic CNP using pairwise *F*_ST_ calculated in the same manner as described previously for SNPs [66]. We also extended the methods of the SNP LSBL analysis to the biallelic CNPs to identify along which branch the greatest changes in allele frequency has occurred using *F*_ST_ to calculate LSBL. Andean-Mesoamerican and Tibetan-East Asian pairwise *F*_ST_ and LSBL values falling within the top 1% of the empirical distribution were identified as statistically significant (data not shown). Next, we implemented a similar technique as that described by Redon to calculate REHH [37]. Each biallelic CNP was treated as a SNP located at the boundary of the CNV window, and SNPs falling 500 kb upstream and downstream of the respective boundary were used to calculate REHH in SWEEP (http://www.broad.mit.edu/mpg/sweep/resources.html). SNPs located within CNPs were excluded. Lastly, we considered rare CNVs identified in Andeans or Tibetans that overlapped specifically with the selection-nominated candidate genes identified from our SNP analysis.

## Supporting Information

Figure S1 Indigenous American ancestry estimates. (A) The effect of admixture on PC plot with all 439,046 SNPs (B) Intermediate PC plot with 240,969 SNPs (threshold 0.9) showing how the gap with the European cluster is reduced. Final step, with threshold 0.8 is shown in Figure 2A (where Europeans (EUR) have been removed to simplify the graphical representation).
(0.27 MB TIF)

Figure S2 Frappe clustering for values of *K* other than that presented in Figure 1C.
(0.70 MB TIF)

Figure S3 Frappe clustering for values of *K* other than that presented in Figure 1D.
(0.74 MB TIF)

Table S1 One megabase windows displaying extended regions of statistical significance calculated using the hypergeometric distribution for LSBL, ln*RH*, and the standardized difference of *D*. All windows listed are statistically significant for the standardized difference of *D* and LSBL or ln*RH* as indicated in column 2.
(0.12 MB DOC)

Table S2 Genes encoded by the four consecutive one megabase windows on chromosome 12 spanning 109,000,000 bp to 113,000,000 bp that were significant for the hypergeometric distribution.
(0.07 MB DOC)

Table S3 The total and significant number of LSBL SNPs, ln*RH* and Tajima's *D* SNP windows for *HIF*, *RAS*, and *globin* candidate genes in Andeans.
(0.20 MB DOC)

Table S4 The total and significant number of LSBL SNPs, ln*RH* and Tajima's *D* SNP windows for *HIF*, *RAS*, and *globin* candidate genes in Tibetans.
(0.20 MB DOC)

Table S5 Significant biallelic CNP LSBLs (p<0.01) in Andeans and Tibetans.
(0.05 MB DOC)

Table S6 Significant Andean CNP REHHs. Tibetans did not display any significant CNP REHHs.
(0.13 MB DOC)

Table S7 CNVs overlapping with extended haplotype homozygosity regions identified by the WGLRH test.
(0.03 MB DOC)

Table S8 Populations assayed using the Affymetrix Inc. (Santa Clara, CA) Genome-Wide Human SNP Array 6.0.
(0.03 MB DOC)
