# Supplementary figures and images for: Identifying Signatures of Natural Selection in Tibetan and Andean Populations Using Dense Genome Scan Data
Source: PLoS Genet. 2010 Sep 9;6(9):e1001116. doi: 10.1371/journal.pgen.1001116 (PMC2936536; doi:10.1371/journal.pgen.1001116)

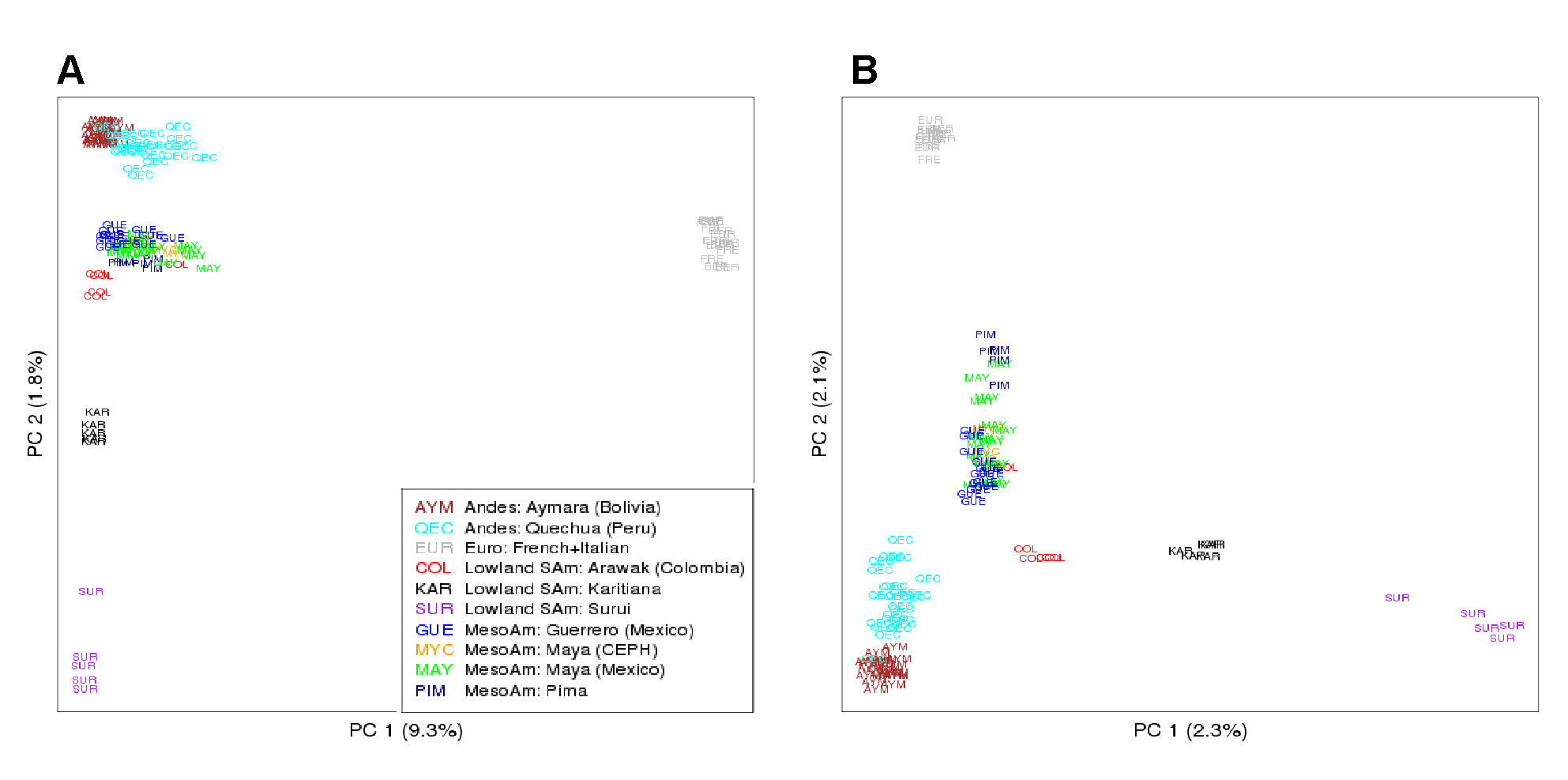

Supplement: Figure S1 — Indigenous American ancestry estimates. (A) The effect of admixture on PC plot with all 439,046 SNPs (B) Intermediate PC plot with 240,969 SNPs (threshold 0.9) showing how the gap with the European cluster is reduced. Final step, with threshold 0.8 is shown in Figure 2A (where Europeans (EUR) have been removed to simplify the graphical representation). (0.27 MB TIF) [file pgen.1001116.s001.tif]

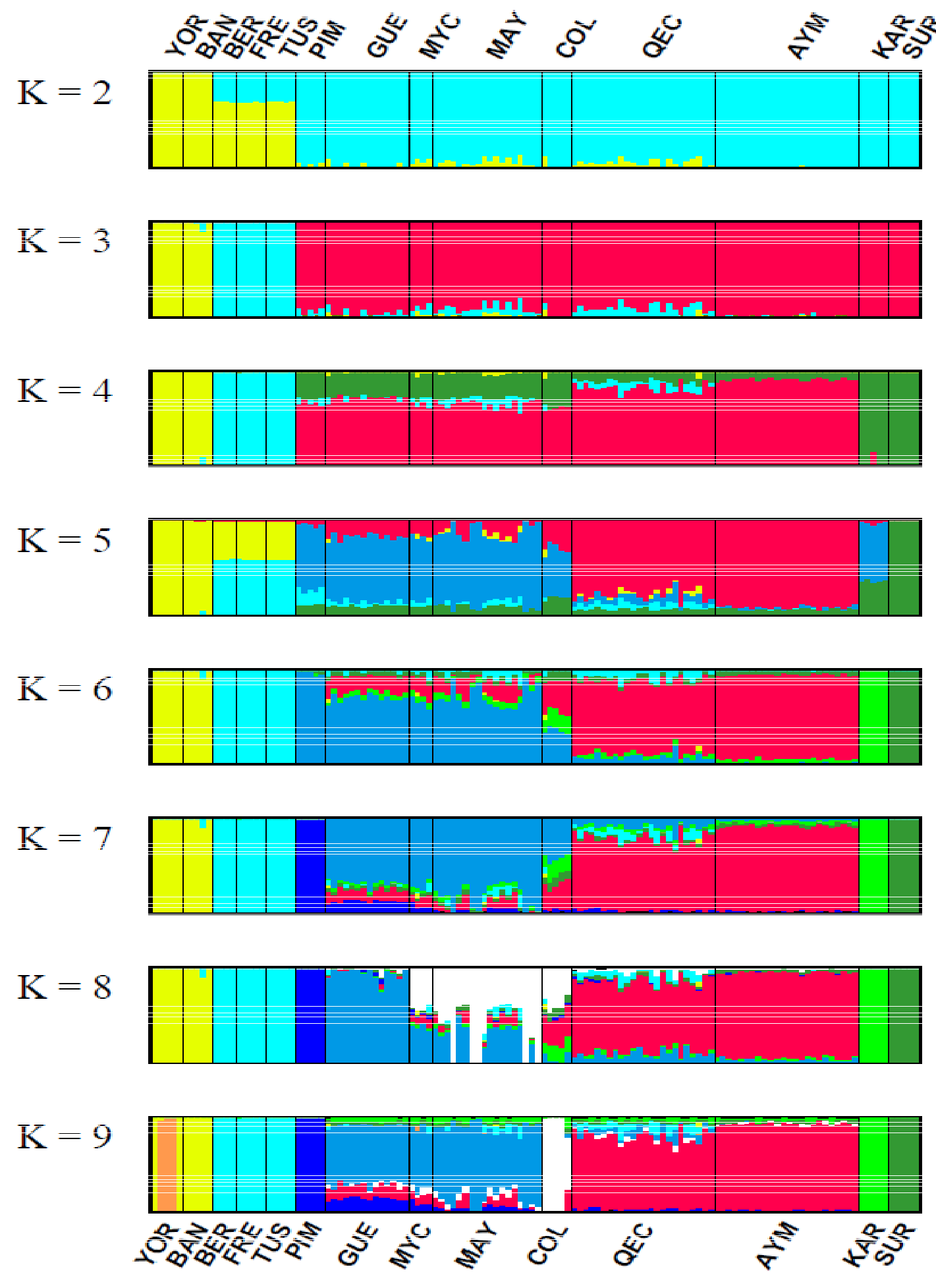

Supplement: Figure S2 — Frappe clustering for values of K other than that presented in Figure 1C. (0.70 MB TIF) [file pgen.1001116.s002.tif]

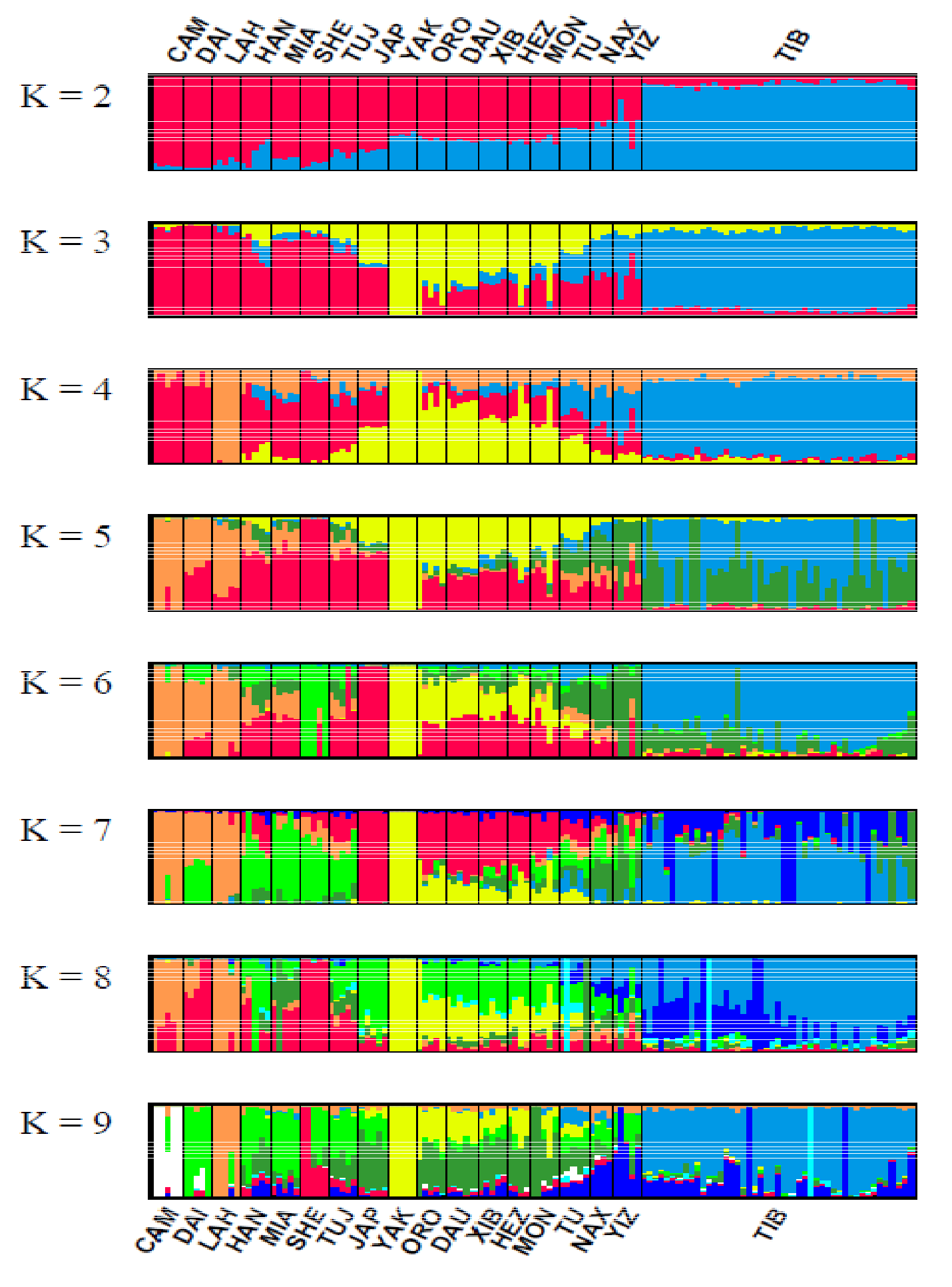

Supplement: Figure S3 — Frappe clustering for values of K other than that presented in Figure 1D. (0.74 MB TIF) [file pgen.1001116.s003.tif]
