# Supplementary material for: Identifying Signatures of Natural Selection in Tibetan and Andean Populations Using Dense Genome Scan Data
Source: PLoS Genet. 2010 Sep 9;6(9):e1001116. doi: 10.1371/journal.pgen.1001116 (PMC2936536; doi:10.1371/journal.pgen.1001116)
Supplement: Table S1 — One megabase windows displaying extended regions of statistical significance calculated using the hypergeometric distribution for LSBL, lnRH, and the standardized difference of D. All windows listed are statistically significant for the standardized difference of D and LSBL or lnRH as indicated in column 2. (0.12 MB DOC) [file pgen.1001116.s004.doc]

**Table S1. One megabase windows displaying extended regions of statistical significance calculated using the hypergeometric distribution for LSBL, ln*RH,* and the standardized difference of *D*. All windows listed are statistically significant for the standardized difference of *D* and LSBL or ln*RH* as indicated in column 2.**

| **Population** | **Statistic** | **Chr** | **Window Start** | **Window End** | **LSBL p-value*** | **ln*RH* p-value*** | **Tajima's *D* p-value*** | **Known Genes** |
| --- | --- | --- | --- | --- | --- | --- | --- | --- |
| Andean | LSBL | 1 | 102000000 | 103000000 | 0.0000 | NA | 0.0010 | 2 |
|  | LSBL | 1 | 188000000 | 189000000 | 0.0000 | NA | 0.0010 | 1 |
|  | LSBL | 2 | 85000000 | 86000000 | 0.0002 | NA | 0.0010 | 19 |
|  | LSBL | 2 | 116000000 | 117000000 | 0.0001 | NA | 0.0000 | 1 |
|  | LSBL | 3 | 69000000 | 70000000 | 0.0000 | NA | 0.0000 | 8 |
|  | LSBL | 3 | 70000000 | 71000000 | 0.0000 | NA | 0.0000 | 1 |
|  | LSBL | 3 | 75000000 | 76000000 | 0.0000 | NA | 0.0000 | 4 |
|  | LSBL | 3 | 147000000 | 148000000 | 0.0022 | NA | 0.0010 | 5 |
|  | LSBL | 4 | 60000000 | 61000000 | 0.0000 | NA | 0.0000 | 0 |
|  | LSBL | 4 | 179000000 | 180000000 | 0.0000 | NA | 0.0000 | 1 |
|  | LSBL | 5 | 102000000 | 103000000 | 0.0000 | NA | 0.0000 | 5 |
|  | LSBL | 6 | 48000000 | 49000000 | 0.0000 | NA | 0.0001 | 1 |
|  | LSBL | 6 | 52000000 | 53000000 | 0.0000 | NA | 0.0000 | 17 |
|  | LSBL | 6 | 157000000 | 158000000 | 0.0000 | NA | 0.0010 | 2 |
|  | LSBL | 7 | 57000000 | 58000000 | 0.0008 | NA | 0.0000 | 3 |
|  | LSBL | 7 | 61000000 | 62000000 | 0.0000 | NA | 0.0003 | 0 |
|  | LSBL | 7 | 64000000 | 65000000 | 0.0000 | NA | 0.0034 | 9 |
|  | LSBL | 7 | 131000000 | 132000000 | 0.0000 | NA | 0.0000 | 1 |
|  | LSBL | 7 | 133000000 | 134000000 | 0.0000 | NA | 0.0001 | 7 |
|  | LSBL & ln*RH* | 8 | 114000000 | 115000000 | 0.0000 | 0.0000 | 0.0000 | 1 |
|  | LSBL | 9 | 35000000 | 36000000 | 0.0000 | NA | 0.0000 | 33 |
|  | LSBL | 11 | 28000000 | 29000000 | 0.0000 | NA | 0.0001 | 3 |
|  | LSBL | 11 | 34000000 | 35000000 | 0.0000 | NA | 0.0000 | 8 |
|  | LSBL | 12 | 37000000 | 38000000 | 0.0000 | NA | 0.0000 | 3 |
|  | LSBL & ln*RH* | 12 | 46000000 | 47000000 | 0.0000 | 0.0010 | 0.0000 | 13 |
|  | LSBL | 12 | 94000000 | 95000000 | 0.0000 | NA | 0.0000 | 11 |
|  | LSBL & ln*RH* | 12 | 109000000 | 110000000 | 0.0000 | 0.0000 | 0.0001 | 15 |
|  | LSBL | 12 | 110000000 | 111000000 | 0.0000 | NA | 0.0000 | 11 |
|  | LSBL | 12 | 111000000 | 112000000 | 0.0000 | NA | 0.0000 | 10 |
|  | LSBL | 12 | 112000000 | 113000000 | 0.0000 | NA | 0.0000 | 13 |
|  | LSBL | 13 | 56000000 | 57000000 | 0.0000 | NA | 0.0000 | 25 |
|  | LSBL | 13 | 87000000 | 88000000 | 0.0000 | NA | 0.0000 | 1 |
|  | LSBL | 14 | 99000000 | 100000000 | 0.0001 | NA | 0.0000 | 15 |
|  | LSBL | 15 | 41000000 | 42000000 | 0.0000 | NA | 0.0000 | 29 |
|  | LSBL | 16 | 28000000 | 29000000 | 0.0000 | NA | 0.0000 | 23 |
|  | ln*RH* | 16 | 46000000 | 47000000 | NA | 0.0010 | 0.0000 | 6 |
|  | LSBL | 17 | 19000000 | 20000000 | 0.0008 | NA | 0.0007 | 16 |
|  | LSBL | 17 | 26000000 | 27000000 | 0.0000 | NA | 0.0000 | 14 |
| Tibetan | LSBL & ln*RH* | 2 | 46000000 | 47000000 | 0.0000 | 0.0000 | 0.0010 | 10 |
|  | LSBL | 2 | 51000000 | 52000000 | 0.0000 | NA | 0.0000 | 1 |
|  | LSBL | 2 | 130000000 | 131000000 | 0.0000 | NA | 0.0002 | 13 |
|  | LSBL | 2 | 178000000 | 179000000 | 0.0006 | NA | 0.0000 | 7 |
|  | LSBL & ln*RH* | 2 | 203000000 | 204000000 | 0.0000 | 0.0010 | 0.0000 | 8 |
|  | LSBL | 3 | 119000000 | 120000000 | 0.0000 | NA | 0.0001 | 0 |
|  | LSBL | 5 | 138000000 | 139000000 | 0.0000 | NA | 0.0000 | 16 |
|  | ln*RH* | 5 | 170000000 | 171000000 | 0.0000 | NA | 0.0000 | 6 |
|  | LSBL | 12 | 33000000 | 34000000 | 0.0000 | NA | 0.0001 | 1 |
|  | LSBL | 12 | 36000000 | 37000000 | 0.0000 | NA | 0.0000 | 1 |
|  | LSBL | 12 | 52000000 | 53000000 | 0.0000 | NA | 0.0000 | 36 |
|  | LSBL | 12 | 118000000 | 119000000 | 0.0000 | NA | 0.0000 | 9 |
|  | LSBL | 22 | 33000000 | 34000000 | 0.0001 | NA | 0.0001 | 2 |
|  | LSBL & ln*RH* | 22 | 39000000 | 40000000 | 0.0000 | 0.0000 | 0.0001 | 15 |
