# Supplementary material for: Identifying Signatures of Natural Selection in Tibetan and Andean Populations Using Dense Genome Scan Data
Source: PLoS Genet. 2010 Sep 9;6(9):e1001116. doi: 10.1371/journal.pgen.1001116 (PMC2936536; doi:10.1371/journal.pgen.1001116)
Supplement: Table S2 — Genes encoded by the four consecutive one megabase windows on chromosome 12 spanning 109,000,000 bp to 113,000,000 bp that were significant for the hypergeometric distribution. (0.07 MB DOC) [file pgen.1001116.s005.doc]

Table S2. Genes encoded by the four consecutive one megabase windows on chromosome 12 spanning 109,000,000 bp to 113,000,000 bp that were significant for the hypergeometric distribution.

| **Gene** | **Full name** |
| --- | --- |
| *ACAD10* | acyl-Coenzyme A dehydrogenase family, member 10 |
| *ALDH2* | aldehyde dehydrogenase 2 family (mitochondrial) |
| *ANAPC7* | anaphase promoting complex subunit 7 |
| *ARPC3* | actin related protein 2/3 complex, subunit 3, 21kDa |
| *ATP2A2* | ATPase, Ca++ transporting, cardiac muscle, slow twitch 2 |
| *ATXN2* | ataxin 2 |
| *BRAP* | BRCA1 associated protein |
| *C12orf24* | chromosome 12 open reading frame 24 |
| *C12orf47* | chromosome 12 open reading frame 47 |
| *C12orf51* | chromosome 12 open reading frame 51 |
| *C12orf52* | chromosome 12 open reading frame 52 |
| *CCDC42B* | coiled-coil domain containing 42B |
| *CCDC63* | coiled-coil domain containing 63 |
| *CUX2* | cut-like homeobox 2 |
| *DDX54* | DEAD (Asp-Glu-Ala-Asp) box polypeptide 54 |
| *DTX1* | deltex homolog 1 (Drosophila) |
| *ERP29* | endoplasmic reticulum protein 29 |
| *FAM109A* | family with sequence similarity 109, member A |
| *GPN3* | GPN-loop GTPase 3 |
| *HVCN1* | hydrogen voltage-gated channel 1 |
| *IFT81* | intraflagellar transport 81 homolog (Chlamydomonas) |
| *IQCD* | IQ motif containing D |
| *LHX5* | LIM homeobox 5 |
| *MAPKAPK5* | mitogen-activated protein kinase-activated protein kinase 5 |
| *MYL2* | myosin, light chain 2, regulatory, cardiac, slow |
| *NAA25* | N(alpha)-acetyltransferase 25, NatB auxiliary subunit |
| *OAS1* | 2',5'-oligoadenylate synthetase 1 |
| *OAS2* | 2',5'-oligoadenylate synthetase 2 |
| *OAS3* | 2',5'-oligoadenylate synthetase 3 |
| *PLBD2* | phospholipase B domain containing 2 |
| *PPP1CC* | protein phosphatase 1, catalytic subunit, gamma isozyme |
| *PPTC7* | PTC7 protein phosphatase homolog (S. cerevisiae) |
| *PTPN11* | protein tyrosine phosphatase, non-receptor type 11 |
| *RAD9B* | RAD9 homolog B (S. pombe) |
| *RASAL1* | RAS protein activator like 1 (GAP1 like) |
| *RBM19* | RNA binding motif protein 19 |
| *RPH3A* | rabphilin 3A homolog (mouse) |
| *RPL6* | ribosomal protein L6 |
| *SDS* | serine dehydratase |
| *SDSL* | serine dehydratase-like |
| *SH2B3* | SH2B adaptor protein 3 |
| *SLC24A6* | solute carrier family 24 (sodium/potassium/calcium exchanger), member 6 |
| *TCTN1* | tectonic family member 1 |
| *TMEM116* | transmembrane protein 116 |
| *TPCN1* | two pore segment channel 1 |
| *TRAFD1* | TRAF-type zinc finger domain containing 1 |
| *VPS29* | vacuolar protein sorting 29 homolog (S. cerevisiae) |
