# Supplementary material for: Identifying Signatures of Natural Selection in Tibetan and Andean Populations Using Dense Genome Scan Data
Source: PLoS Genet. 2010 Sep 9;6(9):e1001116. doi: 10.1371/journal.pgen.1001116 (PMC2936536; doi:10.1371/journal.pgen.1001116)
Supplement: Table S3 — The total and significant number of LSBL SNPs, lnRH and Tajima's D SNP windows for HIF, RAS, and globin candidate genes in Andeans. (0.20 MB DOC) [file pgen.1001116.s006.doc]

Table S3. The total and significant number of LSBL SNPs, ln*RH* and Tajima’s *D* SNP windows for HIF, RAS, and globin candidate genes in Andeans.

| Pathway | Gene | Chr | LSBL | 5% LSBL | ln*RH* | 5% ln*RH* | Tajima's *D* | 5% Tajima's *D* |
| --- | --- | --- | --- | --- | --- | --- | --- | --- |
| HIF | *ARNT* | 1 | 19 | 0 | 11 | 0 | 11 | 0 |
|  | *ATP1A1* | 1 | 1 | 0 | 8 | 2 | 8 | 3 |
|  | *ATP1A2* | 1 | 42 | 2 | 9 | 0 | 9 | 0 |
|  | *ATP1A4* | 1 | 44 | 1 | 9 | 0 | 9 | 0 |
|  | *ATP1B1* | 1 | 67 | 0 | 9 | 0 | 9 | 0 |
|  | *EGLN1* | 1 | 40 | 25 | 11 | 0 | 10 | 2 |
|  | *FRAP1* | 1 | 48 | 0 | 14 | 3 | 14 | 0 |
|  | *PRKAA2* | 1 | 60 | 5 | 10 | 0 | 10 | 0 |
|  | *CXCR4* | 2 | 16 | 6 | 8 | 0 | 8 | 0 |
|  | *EPAS1* | 2 | 107 | 17 | 11 | 0 | 12 | 4 |
|  | *IGFBP2* | 2 | 53 | 5 | 9 | 0 | 9 | 0 |
|  | *IL1A* | 2 | 44 | 0 | 9 | 2 | 9 | 0 |
|  | *IL1B* | 2 | 30 | 0 | 8 | 4 | 9 | 0 |
|  | *NRP2* | 2 | 87 | 4 | 12 | 0 | 12 | 0 |
|  | *TGFA* | 2 | 105 | 17 | 12 | 1 | 13 | 0 |
|  | *CASR* | 3 | 90 | 0 | 12 | 2 | 12 | 0 |
|  | *PIK3CA* | 3 | 34 | 3 | 12 | 0 | 12 | 0 |
|  | *PIK3CB* | 3 | 25 | 0 | 12 | 3 | 12 | 0 |
|  | *TF* | 3 | 72 | 2 | 10 | 1 | 10 | 0 |
|  | *VHL* | 3 | 41 | 0 | 8 | 0 | 9 | 0 |
|  | *EDNRA* | 4 | 68 | 14 | 11 | 1 | 10 | 0 |
|  | *EIF4E* | 4 | 35 | 0 | 10 | 1 | 10 | 0 |
|  | *ELF2* | 4 | 30 | 12 | 13 | 10 | 13 | 0 |
|  | *KDR* | 4 | 37 | 2 | 10 | 0 | 10 | 0 |
|  | *SOD3* | 4 | 45 | 0 | 8 | 0 | 9 | 0 |
|  | *SPRY1* | 4 | 25 | 0 | 8 | 0 | 9 | 0 |
|  | *VEGFC* | 4 | 64 | 1 | 13 | 0 | 12 | 0 |
|  | *ADRA1B* | 5 | 54 | 1 | 10 | 0 | 10 | 0 |
|  | *PRKAA1* | 5 | 29 | 13 | 10 | 0 | 9 | 6 |
|  | *SPRY4* | 5 | 44 | 0 | 9 | 0 | 9 | 0 |
|  | *TERT* | 5 | 29 | 0 | 9 | 0 | 10 | 0 |
|  | *EDN1* | 6 | 51 | 3 | 9 | 0 | 8 | 0 |
|  | *POU5F1* | 6 | 16 | 0 | 8 | 0 | 8 | 0 |
|  | *TNF* | 6 | 32 | 3 | 8 | 3 | 8 | 0 |
|  | *VEGFA* | 6 | 42 | 3 | 9 | 0 | 9 | 0 |
|  | *EPO* | 7 | 8 | 0 | 8 | 0 | 8 | 0 |
|  | *IGFBP1* | 7 | 29 | 3 | 8 | 0 | 8 | 0 |
|  | *IGFBP3* | 7 | 36 | 4 | 9 | 0 | 8 | 0 |
|  | *IL6* | 7 | 61 | 1 | 8 | 0 | 8 | 0 |
|  | *LEP* | 7 | 49 | 0 | 8 | 0 | 8 | 0 |
|  | *PDGFA* | 7 | 0 | 0 | 0 | 0 | 0 | 0 |
|  | *PIK3CG* | 7 | 21 | 0 | 8 | 0 | 6 | 0 |
|  | *COPS5* | 8 | 6 | 3 | 9 | 0 | 9 | 0 |
|  | *RNF139* | 8 | 23 | 0 | 8 | 0 | 8 | 2 |
|  | *SNAI2* | 8 | 27 | 1 | 8 | 0 | 8 | 0 |
|  | *NOTCH1* | 9 | 20 | 2 | 10 | 0 | 10 | 0 |
|  | *TNC* | 9 | 114 | 26 | 12 | 11 | 12 | 0 |
|  | *CUL2* | 10 | 25 | 0 | 11 | 0 | 12 | 0 |
|  | *NRP1* | 10 | 138 | 1 | 14 | 0 | 14 | 1 |
|  | *IGF2* | 11 | 8 | 0 | 1 | 0 | 2 | 0 |
|  | *TH* | 11 | 20 | 2 | 8 | 0 | 8 | 0 |
|  | *VEGFB* | 11 | 11 | 2 | 8 | 0 | 8 | 0 |
|  | *MDM2* | 12 | 38 | 1 | 9 | 0 | 9 | 0 |
|  | *NOS1* | 12 | 97 | 4 | 14 | 0 | 14 | 0 |
|  | *EDNRB* | 13 | 58 | 0 | 11 | 0 | 12 | 5 |
|  | *FLT1* | 13 | 118 | 0 | 16 | 1 | 15 | 0 |
|  | *SPRY2* | 13 | 44 | 2 | 10 | 0 | 10 | 3 |
|  | *EGLN3* | 14 | 49 | 2 | 9 | 0 | 9 | 0 |
|  | *HIF1A* | 14 | 24 | 0 | 10 | 1 | 10 | 0 |
|  | *PGF* | 14 | 18 | 1 | 8 | 0 | 9 | 0 |
|  | *ARNT2* | 15 | 111 | 15 | 16 | 0 | 15 | 0 |
|  | *CDH1* | 16 | 55 | 1 | 12 | 0 | 12 | 3 |
|  | *MMP2* | 16 | 78 | 7 | 9 | 0 | 9 | 0 |
|  | *SNAI3* | 16 | 22 | 2 | 9 | 0 | 9 | 0 |
|  | *TSC2* | 16 | 8 | 0 | 9 | 0 | 9 | 1 |
|  | *BECN1* | 17 | 8 | 0 | 9 | 0 | 8 | 0 |
|  | *NOS2A* | 17 | 40 | 7 | 10 | 0 | 9 | 4 |
|  | *POLR2A* | 17 | 31 | 5 | 9 | 7 | 10 | 3 |
|  | *EGLN2* | 19 | 29 | 5 | 9 | 0 | 8 | 1 |
|  | *HIF3A* | 19 | 29 | 0 | 10 | 0 | 10 | 0 |
|  | *JUNB* | 19 | 10 | 0 | 8 | 3 | 8 | 0 |
|  | *TGFB1* | 19 | 16 | 0 | 9 | 0 | 9 | 0 |
|  | *PDGF2* | 22 | 30 | 0 | 9 | 0 | 9 | 0 |
|  | *RBX1* | 22 | 14 | 0 | 8 | 0 | 9 | 0 |
|  | *ARD1A* | 23 | 19 | 0 | 9 | 0 | 8 | 0 |
| RAS | *AGT* | 1 | 59 | 0 | 4 | 0 | 5 | 0 |
|  | *AGTRAP* | 1 | 35 | 5 | 5 | 3 | 5 | 0 |
|  | *REN* | 1 | 59 | 0 | 5 | 0 | 4 | 1 |
|  | *AGTR1* | 3 | 89 | 0 | 6 | 0 | 6 | 0 |
|  | *NOS3* | 7 | 19 | 0 | 5 | 0 | 5 | 0 |
|  | *AGTRL-1* | 11 | 40 | 0 | 4 | 0 | 4 | 0 |
|  | *ACE* | 17 | 30 | 1 | 6 | 0 | 5 | 0 |
|  | *ACEII* | X | 29 | 0 | 6 | 0 | 5 | 0 |
|  | *AGTR2* | X | 26 | 2 | 4 | 0 | 4 | 0 |
|  | *ATP6AP2* | X | 27 | 0 | 5 | 5 | 5 | 0 |
|  | *RENBP* | X | 18 | 0 | 4 | 0 | 5 | 0 |
| Globin | *NR2C2* | 3 | 47 | 2 | 7 | 0 | 8 | 0 |
|  | *SATB1* | 3 | 41 | 1 | 7 | 0 | 7 | 2 |
|  | *MAFK* | 7 | 16 | 0 | 4 | 0 | 5 | 0 |
|  | *ZHX2* | 8 | 139 | 0 | 12 | 0 | 12 | 0 |
|  | *ANKRD49* | 11 | 54 | 1 | 4 | 0 | 4 | 0 |
|  | *HBB* | 11 | 64 | 10 | 4 | 0 | 4 | 0 |
|  | *HBD* | 11 | 63 | 6 | 8 | 0 | 8 | 0 |
|  | *HBE1* | 11 | 261 | 35 | 18 | 0 | 17 | 0 |
|  | *HBG1* | 11 | 60 | 0 | 8 | 0 | 8 | 0 |
|  | *HBG2* | 11 | 56 | 0 | 8 | 0 | 8 | 0 |
|  | *NFE2* | 12 | 5 | 0 | 9 | 3 | 8 | 0 |
|  | *NFE4* | 12 | 42 | 0 | 8 | 0 | 7 | 0 |
|  | *NR2C1* | 12 | 37 | 0 | 6 | 0 | 6 | 2 |
|  | *SP1* | 12 | 18 | 0 | 5 | 0 | 5 | 0 |
|  | *TFCP2* | 12 | 42 | 0 | 12 | 0 | 11 | 0 |
|  | *NGB* | 14 | 50 | 4 | 4 | 0 | 5 | 0 |
|  | *C16orf35* | 16 | 35 | 4 | 8 | 4 | 7 | 0 |
|  | *HBA1* | 16 | 11 | 3 | 4 | 0 | 4 | 0 |
|  | *HBA2* | 16 | 10 | 2 | 8 | 4 | 8 | 0 |
|  | *HBQ1* | 16 | 11 | 3 | 8 | 4 | 8 | 0 |
|  | *HBZ* | 16 | 17 | 1 | 8 | 4 | 7 | 0 |
|  | *MPG* | 16 | 32 | 3 | 6 | 4 | 4 | 0 |
|  | *CYGB* | 17 | 44 | 5 | 5 | 0 | 5 | 0 |
|  | *KLF2* | 19 | 23 | 1 | 4 | 0 | 4 | 0 |
|  | *MB* | 22 | 44 | 0 | 4 | 0 | 5 | 4 |
|  | *GATA1* | X | 6 | 0 | 4 | 0 | 8 | 0 |
|  | *KLF8* | X | 12 | 1 | 6 | 5 | 10 | 0 |
