# Supplementary material for: Identifying Signatures of Natural Selection in Tibetan and Andean Populations Using Dense Genome Scan Data
Source: PLoS Genet. 2010 Sep 9;6(9):e1001116. doi: 10.1371/journal.pgen.1001116 (PMC2936536; doi:10.1371/journal.pgen.1001116)
Supplement: Table S5 — Significant biallelic CNP LSBLs (p<0.01) in Andeans and Tibetans. (0.05 MB DOC) [file pgen.1001116.s008.doc]

**Table S5.** Significant biallelic CNP LSBLs (p<0.01) in Andeans and Tibetans.

| **Population** | **CNP ID** | **Chromosome** | **CNP Start** | **CNP End** | **LSBL** | **Known Genes** |
| --- | --- | --- | --- | --- | --- | --- |
| Andean | CNP876 | 5 | 151495579 | 151498544 | 0.20 | None |
|  | CNP12448 | 16 | 58640103 | 58654487 | 0.15 | None |
|  | CNP1115 | 7 | 86072821 | 86082341 | 0.14 | None |
|  | CNP10982 | 5 | 143386880 | 143390529 | 0.13 | None |
|  | CNP160 | 1 | 213560092 | 213565727 | 0.08 | None |
|  | CNP11449 | 8 | 36194697 | 36197883 | 0.07 | None |
|  | CNP10188 | 1 | 193280600 | 193285922 | 0.07 | None |
| Tibetan | CNP10282 | 2 | 46549602 | 46551188 | 0.53 | None |
|  | CNP790 | 5 | 38180803 | 38184641 | 0.26 | None |
|  | CNP2368 | 19 | 8256197 | 8271067 | 0.22 | None |
|  | CNP1522 | 9 | 133250747 | 133255525 | 0.13 | None |
|  | CNP10815 | 4 | 153209736 | 153212189 | 0.09 | None |
|  | CNP11826 | 11 | 5842285 | 5892086 | 0.08 | *OR52E4* |
|  | CNP1395 | 9 | 4518770 | 4519873 | 0.07 | *SLC1A1* |
