# Supplementary material for: Identifying Signatures of Natural Selection in Tibetan and Andean Populations Using Dense Genome Scan Data
Source: PLoS Genet. 2010 Sep 9;6(9):e1001116. doi: 10.1371/journal.pgen.1001116 (PMC2936536; doi:10.1371/journal.pgen.1001116)
Supplement: Table S6 — Significant Andean CNP REHHs. Tibetans did not display any significant CNP REHHs. (0.13 MB DOC) [file pgen.1001116.s009.doc]

**Table S6. Significant Andean CNP REHHs. Tibetans did not display any significant CNP REHHs.**

| **Core Source** | **Chr** | **CNV Start** | **CNV End** | **Haplotype Frequency** | **EHH** | **REHH** | **EHH p-value** | **REHH p-value** | **Genes In Region** |
| --- | --- | --- | --- | --- | --- | --- | --- | --- | --- |
| CNP160 | 1 | 213560092 | 213565727 | 0.806 | 0.070 | 1.989 | 0.034 | 0.029 | NULL |
| CNP10387 | 2 | 133189998 | 133193035 | 0.918 | 0.040 | 1.133 | 0.572 | 0.036 | *NCKAP5* |
| CNP10437 | 2 | 205366265 | 205391991 | 0.939 | 0.066 | 0.985 | 0.014 | 0.048 | *PARD3B* |
| CNP10458 | 3 | 1009997 | 1014189 | 0.918 | 0.073 | 2.048 | 0.003 | 0.009 | NULL |
| CNP10510 | 3 | 41755151 | 41763499 | 0.929 | 0.057 | 1.195 | 0.069 | 0.033 | *ULK4* |
| CNP10538 | 3 | 77910146 | 77924562 | 0.929 | 0.064 | 1.338 | 0.021 | 0.026 | NULL |
| CNP519 | 3 | 174692475 | 174698428 | 0.918 | 0.044 | 1.230 | 0.412 | 0.031 | NLGN1 |
| CNP10700 | 4 | 43433755 | 43436627 | 0.918 | 0.048 | 1.356 | 0.243 | 0.025 | NULL |
| CNP10709 | 4 | 57951132 | 57953111 | 0.857 | 0.067 | 6.056 | 0.054 | 0.001 | NULL |
| CNP10718 | 4 | 65502562 | 65522613 | 0.908 | 0.057 | 2.059 | 0.066 | 0.009 | NULL |
| CNP10720 | 4 | 66271146 | 66312360 | 0.918 | 0.048 | 1.342 | 0.259 | 0.025 | NULL |
| CNP10743 | 4 | 78001593 | 78006231 | 0.918 | 0.047 | 1.328 | 0.276 | 0.026 | NULL |
| CNP620 | 4 | 87195118 | 87198968 | 0.929 | 0.047 | 0.985 | 0.295 | 0.048 | *MAPK10* |
| CNP10760 | 4 | 97539253 | 97541426 | 0.867 | 0.048 | 3.714 | 0.470 | 0.004 | NULL |
| CNP10784 | 4 | 121743029 | 121762869 | 0.929 | 0.077 | 1.615 | 0.002 | 0.017 | NULL |
| CNP10804 | 4 | 144938147 | 144965448 | 0.929 | 0.052 | 1.087 | 0.153 | 0.039 | NULL |
| CNP704 | 4 | 156813718 | 156817445 | 0.918 | 0.045 | 1.251 | 0.380 | 0.030 | *GUCY1A3* |
| CNP753 | 5 | 8755523 | 8800138 | 0.929 | 0.056 | 1.179 | 0.078 | 0.033 | NULL |
| CNP10896 | 5 | 17400702 | 17409239 | 0.898 | 0.046 | 2.069 | 0.534 | 0.026 | NULL |
| CNP790 | 5 | 38180803 | 38184641 | 0.918 | 0.070 | 0.986 | 0.006 | 0.048 | NULL |
| CNP10946 | 5 | 97961571 | 97963403 | 0.898 | 0.049 | 2.222 | 0.405 | 0.021 | NULL |
| CNP839 | 5 | 101201120 | 101234788 | 0.918 | 0.061 | 1.720 | 0.032 | 0.014 | NULL |
| CNP846 | 5 | 104464614 | 104506104 | 0.918 | 0.046 | 1.279 | 0.340 | 0.028 | NULL |
| CNP10955 | 5 | 106257572 | 106261016 | 0.898 | 0.048 | 2.151 | 0.463 | 0.023 | NULL |
| CNP10964 | 5 | 114734342 | 114748984 | 0.918 | 0.047 | 1.328 | 0.276 | 0.026 | NULL |
| CNP10981 | 5 | 141998202 | 142000197 | 0.929 | 0.048 | 1.015 | 0.245 | 0.045 | *FGF1* |
| CNP11021 | 6 | 10175992 | 10177866 | 0.918 | 0.053 | 1.482 | 0.129 | 0.020 | NULL |
| CNP11043 | 6 | 31467630 | 31559451 | 0.827 | 0.069 | 9.360 | 0.039 | 0.000 | *MICA, HCP5, HCG26* |
| CNP11104 | 6 | 82203843 | 82228325 | 0.918 | 0.043 | 1.202 | 0.456 | 0.032 | NULL |
| CNP11134 | 6 | 132751050 | 132754736 | 0.918 | 0.045 | 1.258 | 0.370 | 0.029 | *MOXD1* |
| CNP11145 | 6 | 147252374 | 147261510 | 0.929 | 0.051 | 1.072 | 0.170 | 0.041 | NULL |
| CNP11169 | 6 | 168718729 | 168720559 | 0.908 | 0.070 | 1.264 | 0.006 | 0.029 | *SMOC2* |
| CNP11176 | 7 | 3400802 | 3405749 | 0.929 | 0.051 | 1.062 | 0.183 | 0.041 | SDK1 |
| CNP11221 | 7 | 48552398 | 48562571 | 0.918 | 0.050 | 1.398 | 0.199 | 0.023 | *ABCA13* |
| CNP11222 | 7 | 49144690 | 49148378 | 0.878 | 0.049 | 1.625 | 0.409 | 0.048 | NULL |
| CNP11424 | 8 | 14832796 | 14840174 | 0.908 | 0.049 | 1.747 | 0.240 | 0.014 | *SGCZ* |
| CNP1294 | 8 | 40302954 | 40308668 | 0.918 | 0.049 | 1.363 | 0.235 | 0.025 | NULL |
| CNP1320 | 8 | 68710013 | 68712760 | 0.939 | 0.066 | 0.996 | 0.013 | 0.047 | *CPA6* |
| CNP11501 | 8 | 107927695 | 107931446 | 0.908 | 0.050 | 1.793 | 0.203 | 0.013 | NULL |
| CNP11510 | 8 | 117699864 | 117702442 | 0.898 | 0.077 | 3.444 | 0.012 | 0.005 | NULL |
| CNP11524 | 8 | 136692574 | 136694147 | 0.908 | 0.042 | 1.526 | 0.481 | 0.019 | *KHDRBS3* |
| CNP11533 | 9 | 139481 | 264606 | 0.918 | 0.048 | 1.335 | 0.267 | 0.026 | *CBWD1, LOC642313, C9orf66, DOCK8* |
| CNP1395 | 9 | 4518770 | 4519873 | 0.847 | 0.051 | 1.769 | 0.364 | 0.039 | *SLC1A1* |
| CNP11545 | 9 | 5301567 | 5327707 | 0.898 | 0.063 | 2.845 | 0.085 | 0.010 | *RLN1* |
| CNP11549 | 9 | 6662095 | 6663766 | 0.918 | 0.058 | 1.636 | 0.053 | 0.016 | NULL |
| CNP11577 | 9 | 24487680 | 24498238 | 0.929 | 0.055 | 1.159 | 0.091 | 0.035 | NULL |
| CNP1495 | 9 | 106403809 | 106406604 | 0.908 | 0.052 | 1.857 | 0.158 | 0.012 | NULL |
| CNP12448 | 16 | 58640103 | 58654487 | 0.276 | 0.199 | 5.763 | 0.143 | 0.050 | NULL |
| CNP2200 | 16 | 74115584 | 74133500 | 0.929 | 0.060 | 1.262 | 0.040 | 0.029 | *CHST5, TMEM231* |
| CNP12616 | 18 | 74763700 | 74765854 | 0.888 | 0.055 | 3.043 | 0.223 | 0.008 | NULL |
| CNP12674 | 19 | 53881228 | 53897953 | 0.908 | 0.054 | 0.970 | 0.112 | 0.049 | *FUT2* |
| CNP12770 | 21 | 31354121 | 31356175 | 0.929 | 0.049 | 1.026 | 0.230 | 0.044 | NULL |
| CNP12774 | 21 | 39656792 | 39658882 | 0.908 | 0.040 | 1.434 | 0.601 | 0.022 | NULL |
| CNP12781 | 21 | 45599902 | 45603200 | 0.918 | 0.060 | 1.671 | 0.043 | 0.015 | NULL |
| CNP12788 | 22 | 17006129 | 17009149 | 0.918 | 0.043 | 1.202 | 0.456 | 0.032 | NULL |
