# Supplementary material for: Identifying Signatures of Natural Selection in Tibetan and Andean Populations Using Dense Genome Scan Data
Source: PLoS Genet. 2010 Sep 9;6(9):e1001116. doi: 10.1371/journal.pgen.1001116 (PMC2936536; doi:10.1371/journal.pgen.1001116)
Supplement: Table S7 — CNVs overlapping with extended haplotype homozygosity regions identified by the WGLRH test. (0.03 MB DOC) [file pgen.1001116.s010.doc]

**Table S7.** CNVs overlapping with extended haplotype homozygosity regions identified by the WGLRH test.

| Chromosome | CNV Start | CNV End | Extended Haplotype Start | Extended Haplotype End | Haplotype Frequency | p-value Adjusted | Population |
| --- | --- | --- | --- | --- | --- | --- | --- |
| 3 | 3648608 | 3703697 | 3660379 | 3662709 | 0.255 | 0.0035 | Tibetan |
| 13 | 42277500 | 42719247 | 42630704 | 42631457 | 0.418 | 0.0389 | Tibetan |
| 18 | 27903953 | 27931381 | 27919710 | 27919861 | 0.306 | 0.0300 | Tibetan |
| 1 | 167909246 | 167956424 | 167935872 | 167937741 | 0.496 | 0.0066 | Andean |
