# Supplementary material for: Identifying Signatures of Natural Selection in Tibetan and Andean Populations Using Dense Genome Scan Data
Source: PLoS Genet. 2010 Sep 9;6(9):e1001116. doi: 10.1371/journal.pgen.1001116 (PMC2936536; doi:10.1371/journal.pgen.1001116)
Supplement: Table S8 — Populations assayed using the Affymetrix Inc. (Santa Clara, CA) Genome-Wide Human SNP Array 6.0. (0.03 MB DOC) [file pgen.1001116.s011.doc]

**Table S8.** Populations assayed using the Affymetrix Inc. (Santa Clara, CA) Genome-Wide Human SNP Array 6.0.

| **Population** | **Sample Size (n)** |
| --- | --- |
| Tibetan | 49 |
| Quechua | 24 |
| Aymara | 25 |
| Mexican* | 14 |
| Mayan | 25 |
| East Asian | 90 |
| European | 60 |
| Yoruba | 60 |
| *Mixtec, Tlapanec, and Nahua Speakers | |
